# A Small Molecule Glycosaminoglycan Mimetic Blocks *Plasmodium* Invasion of the Mosquito Midgut

**DOI:** 10.1371/journal.ppat.1003757

**Published:** 2013-11-21

**Authors:** Derrick K. Mathias, Rebecca Pastrana-Mena, Elisabetta Ranucci, Dingyin Tao, Paolo Ferruti, Corrie Ortega, Gregory O. Staples, Joseph Zaia, Eizo Takashima, Takafumi Tsuboi, Natalie A. Borg, Luisella Verotta, Rhoel R. Dinglasan

**Affiliations:** 1 W. Harry Feinstone Department of Molecular Microbiology and Immunology, Malaria Research Institute, Johns Hopkins Bloomberg School of Public Health, Baltimore, Maryland, United States of America; 2 Department of Chemistry, University of Milan, Milan, Italy; 3 Department of Biochemistry and Center for Biomedical Mass Spectrometry, Boston University School of Medicine, Boston, Massachusetts, United States of America; 4 Division of Malaria Research, Proteo-Science Center, Ehime University, Matsuyama, Ehime, Japan; 5 Department of Biochemistry and Molecular Biology, School of Biomedical Sciences, Monash University, Clayton, Victoria, Australia; Stanford University, United States of America

## Abstract

Malaria transmission-blocking (T-B) interventions are essential for malaria elimination. Small molecules that inhibit the *Plasmodium* ookinete-to-oocyst transition in the midgut of *Anopheles* mosquitoes, thereby blocking sporogony, represent one approach to achieving this goal. Chondroitin sulfate glycosaminoglycans (CS-GAGs) on the *Anopheles gambiae* midgut surface are putative ligands for *Plasmodium falciparum* ookinetes. We hypothesized that our synthetic polysulfonated polymer, VS1, acting as a decoy molecular mimetic of midgut CS-GAGs confers malaria T-B activity. In our study, VS1 repeatedly reduced midgut oocyst development by as much as 99% (*P*<0.0001) in mosquitoes fed with *P. falciparum* and *Plasmodium berghei*. Through direct-binding assays, we observed that VS1 bound to two critical ookinete micronemal proteins, each containing at least one von Willebrand factor A (vWA) domain: (i) circumsporozoite protein and thrombospondin-related anonymous protein-related protein (CTRP) and (ii) vWA domain-related protein (WARP). By immunofluorescence microscopy, we observed that VS1 stains permeabilized *P. falciparum* and *P. berghei* ookinetes but does not stain *P. berghei* CTRP knockouts or transgenic parasites lacking the vWA domains of CTRP while retaining the thrombospondin repeat region. We produced structural homology models of the first vWA domain of CTRP and identified, as expected, putative GAG-binding sites on CTRP that align closely with those predicted for the human vWA A1 domain and the *Toxoplasma gondii* MIC2 adhesin. Importantly, the models also identified patches of electropositive residues that may extend CTRP's GAG-binding motif and thus potentiate VS1 binding. Our molecule binds to a critical, conserved ookinete protein, CTRP, and exhibits potent malaria T-B activity. This study lays the framework for a high-throughput screen of existing libraries of safe compounds to identify those with potent T-B activity. We envision that such compounds when used as partner drugs with current antimalarial regimens and with RTS,S vaccine delivery could prevent the transmission of drug-resistant and vaccine-breakthrough strains.

## Author Summary

To achieve malaria elimination, the consensus expert opinion is that new approaches to drug and vaccine design are desperately needed. We have undertaken a novel, comprehensive approach towards the development of a malaria transmission-blocking drug based on the strategy of inhibiting *Plasmodium* development in the mosquito by interfering with obligate cellular interactions between the parasite and the mosquito-midgut epithelium. We have successfully designed a potent transmission-blocking small molecule (VS1) that mimics the structure of molecules on the mosquito-midgut surface called glycosaminoglycans (GAG), which are thought to serve as ligands for parasite attachment prior to cell invasion. Using assays in which mosquitoes were fed with infectious blood, we tested the effect of VS1 on *Plasmodium* development in the mosquito and found that the GAG mimic dramatically reduced the intensity of infection in the midgut. Binding experiments and immunofluorescence microscopy indicate that VS1 binds to the circumsporozoite- and TRAP-related protein (CTRP), a micronemal protein expressed by ookinetes essential for midgut invasion. This interaction profoundly inhibits a key step of parasite development, thereby abrogating downstream events necessary for mosquito-to-human transmission. The work described lays the framework for bringing a truly novel transmission-blocking drug to fruition.

## Introduction

Each year more than half a million people die from malaria, a disease caused by protozoan parasites in the genus *Plasmodium*. The life cycle of *Plasmodium* parasites includes asexual development in the human host and obligatory sporogonic development in the *Anopheles* mosquito vector with transmission from person to person only made possible through the bite of an infected anopheline. Despite substantial investment in malaria research, it is widely accepted that current interventions are insufficient to achieve the ultimate goal of eradication and that a combination of anti-malaria strategies including those that target parasite transmission give eradication efforts the best chance to succeed [1]. Moreover, the evolutionary capacity of vectors and parasites to overcome chemical- and drug-based interventions emphasizes the need for new weapons in the anti-malaria arsenal. It is in this context that malaria transmission-blocking (T-B) interventions (vaccines and drugs) have received significant attention [2]. In fact, recent progress has shown that probing the basic biology underlying mosquito-*Plasmodium* interactions can identify novel intervention targets not only in the parasite, but in the mosquito as well [3]–[6]. Importantly, seminal work by Delves, *et al*. [7] have brought increased attention to the potential T-B activity of drugs that failed to demonstrate efficacy against *Plasmodium* asexual stages but have been resurrected as novel T-B candidate compounds. These efforts highlight the need for T-B molecules and open new avenues for the development and/or repurposing of compounds that have direct activity against the parasite soon after ingestion into the mosquito midgut during blood feeding.

In the mosquito blood meal, *Plasmodium* gametocytes differentiate into gametes and fuse to form zygotes, which then develop into motile ookinetes. For parasite development to continue, ookinetes must find and adhere to membrane-associated ligands on the midgut epithelial surface, a pre-requisite for cell invasion. Experimental evidence from *Plasmodium berghei* and *Plasmodium falciparum* suggests that ookinete attachment and invasion is mediated by micronemal proteins, including the circumsporozoite protein and thrombospondin-related anonymous protein-related protein (CTRP) [8]–[13] and von Willebrand factor A domain-related protein (WARP) [10], [14]. The function of WARP is unclear, while CTRP has a demonstrated role in ookinete motility [9], [15]. However, both are essential for midgut epithelial cell invasion by *Plasmodium* ookinetes. Once inside the cell, ookinetes make their way to the midgut basal lamina where they differentiate into oocysts, each giving rise to thousands of sporozoites that are released into the hemocoel upon maturation and rupture. Sporozoites are then swept into the circulating hemolymph and carried to the salivary glands. Following successful salivary gland invasion, sporozoites remain in the lumen of the salivary duct until host delivery during blood feeding. Clearly, negotiating the midgut tissue barrier in the vector is crucial for successful establishment of the parasite in the mosquito and hence, subsequent transmission to human hosts.

**Figure 1:**
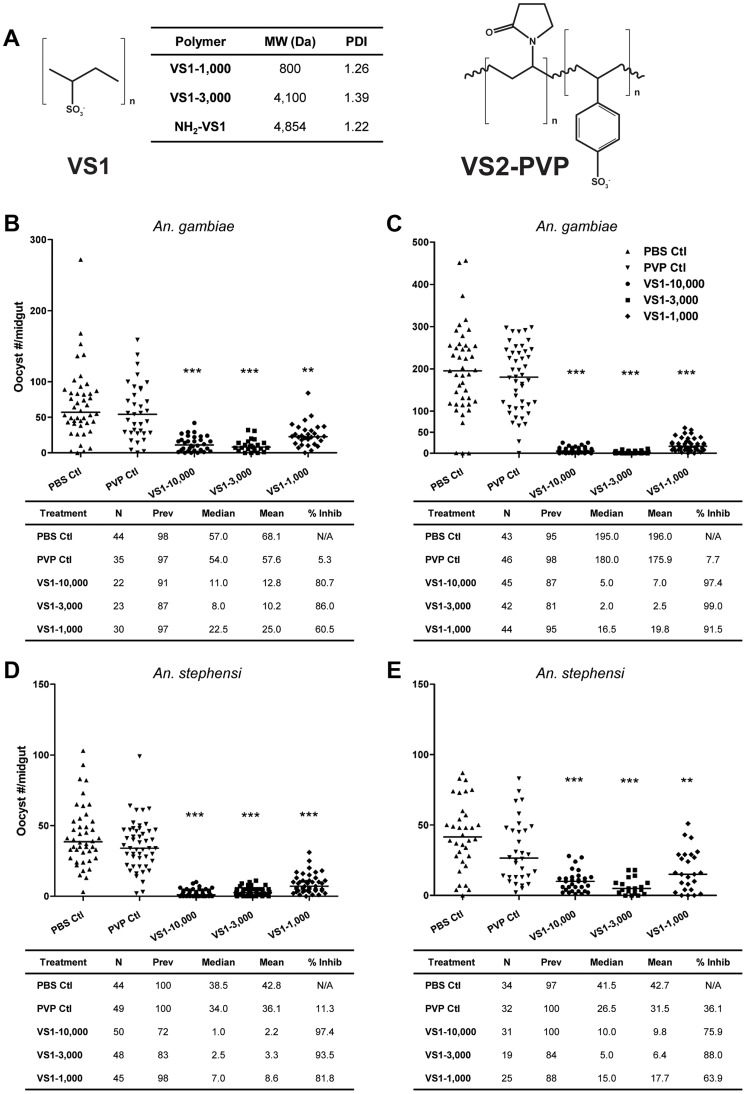
VS1 exhibits marked transmission-blocking activity against *P. falciparum* in two divergent anopheline species. (**A**) Structure of compounds designed to interfere with ionic interactions between *Plasmodium* ookinetes and midgut apical-surface associated glycosaminoglycans (Figure S1A). The table indicates the average molecular weight (MW) and polydispersity index (PDI) for each species of compound tested in the study. (**B–C**) Results from representative replicate SMFAs showing *P. falciparum* midgut-oocyst intensities in *An. gambiae* plotted by treatment group. Below each graph, experimental details are provided, where N is the number of mosquito midguts dissected and scored for oocysts, prev is the infection prevalence among mosquitoes in a given treatment group, median is the median oocyst number, mean is the mean oocyst number, and % inhibition is calculated as (median_control_ – median_treatment_)/median_control_. (**D–E**) Same as panels B–C except that *An. stephensi* were used. The level of statistical significance is denoted by asterisks following Bonferroni correction of *z-*scores, * = p<0.05, ** = p<0.01, *** = p<0.001.

In this study we exploit knowledge of crucial molecular interactions between *Plasmodium* ookinetes and the apical microvillar surface of the mosquito midgut to design proof-of-concept small molecules that interfere with ookinete attachment. Previous work demonstrated that sulfated glycosaminoglycans (GAGs) are present on both the apical and basal surfaces of the midgut epithelium, with chondroitin sulfate (CS) predominant on the apical side (i.e., facing the midgut lumen) and heparan sulfate (HS) predominant on the basal side [5], [16] (Figure S1A). RNAi-mediated knockdown of the *Anopheles gambiae* peptide-*O-*xylosyltransferase, an enzyme that catalyzes the first step in CS and HS biosynthesis, resulted in mosquitoes with CS-depleted midgut apical surfaces [5]. When infected with *P. falciparum* and *P. berghei* in feeding assays, these mosquitoes demonstrated significantly lower oocyst infection intensities relative to controls. The study also showed binding affinity for two types of CS (CS-A and CS-E) in mature ookinetes, consistent with a previous report demonstrating that the ookinete micronemal proteins PfWARP and PfCTRP bind to sulfated GAGs *in vitro* [10]. Inspired by the idea that these molecular interactions could be disrupted by small molecules that mimic the charged structural elements involved in ligand binding, two short-chain, water-soluble compounds were synthesized for *in vitro* and *in vivo* T-B studies based on their potential to interfere with parasite protein-GAG interactions (Figure 1A).

Here we describe efforts to test this strategy with the underlying hypothesis that when a mosquito ingests these small molecules in an infectious blood meal, the compounds will interfere with ookinete-GAG interactions, therefore preventing midgut invasion and abrogating subsequent developmental steps in sporogony. Based on key studies in the literature [9]–[15], we further hypothesize that the molecular basis of ookinete-GAG interactions, and hence those between ookinetes and our GAG-mimetics, involve the *Plasmodium* micronemal proteins CTRP and/or WARP. Our findings showed that this novel strategy dramatically reduced infection intensity in the mosquito midgut, and multiple lines of evidence suggest that the mechanism underlying the T-B effect involved binding of our GAG-mimetic decoy to one or more von Willebrand factor A (vWA) domains found in the protein CTRP.

## Materials and Methods

### Synthesis of small molecule inhibitors

Synthesis of polysulfonated polymers (VS1 and VS2-PVP) proceeded with the addition of 100 mg of potassium persulfate to 5 g (0.38 mmol) of vinyl-sulfonic acid (VS1) sodium salt water solution and adjusted to basic pH with sodium hydroxide. The final solution was warmed for 20 hr at 80°C, then cooled to room temperature (RT), diluted with water and ultra-filtered through a membrane with a nominal cut-off of 10,000 Da. The fraction retained was freeze-dried and the product obtained was a white powder. Size exclusion chromatography was used following different reaction times to obtain oligomers of different length and molecular mass. These compounds were then purified by ultra-filtration through different cut-off membranes (500 Da, 1000 Da, 5000 Da), and average molecular weights were measured by size exclusion chromatography and MALDI spectrometry.

### Parasites, mosquito maintenance and transmission-blocking assays

Transmission-blocking assays for vinyl-sulfonic acid compounds (including preliminary VS1 and VS2-PVP experiments) were tested using both *in vivo* and *in vitro* systems. *In vivo* studies were performed using the murine malaria parasite *P. berghei* (ANKA 2.34) following IACUC approved protocols. For each experiment, two to three naïve, donor mice (Swiss Webster, 20–24 g) were inoculated (i.v.) with blood stage *P. berghei* and then checked for parasitemia by blood smear five to six days later. Once parasitemia reached ≥10%, donors were sacrificed via cardiac puncture and parasitemic blood was used to inoculate (i.v.) eight to ten experimental mice per test compound. Two to three days post-inoculation, experimental mice were smeared and checked for exflagellating gametocytes. Mice demonstrating an average of at least 1 and fewer than 6 exflagellations per 40× field were assigned to a treatment group, weighed and anesthetized. For each mouse, a pre-injection 500 ml cup of *Anopheles stephensi* mosquitoes (*n* = 50) were allowed to feed for 15 to 20 min. The mouse was then removed from the mosquito cup, injected with either a vinyl sulfonic acid compound (250 µg/ml or 500 µg per 24 g body weight), polyvinylpyrrolidone (PVP, same dose as VS1or VS2-PVP), or the carrier only (PBS) via tail vein injection (iv) and then allowed to recover for 10 to 15 min. Following recovery, a post-injection cup of mosquitoes (*n* = 50) was allowed to feed as before. Unfed mosquitoes were then removed from both pre- and post-injection cups via mouth aspiration. For each control and test compound, three to five pre- and post-injection sets of mosquitoes were maintained on sucrose and water for 10 days at 19°C, 80% relative humidity. On day 10, midguts were dissected from all surviving mosquitoes and stained with 0.1% mercurochrome for 20 mins. Oocyst number for each midgut was determined by microscopy and at least three independent experiments were performed for each compound.

*In vitro* studies were performed using the human malaria parasite *P. falciparum* and the old-world malaria vectors *Anopheles gambiae* and *An. stephensi as* described [4]–[5]. With the exception of the parasite load experiment, each set of studies consisted of independent experiments in which the age of the gametocyte culture (16–17 days), the age of mosquitoes (4–6 days), and the blood-meal gametocytemia (0.3%) and hematocrit (45%) were kept consistent. In the parasite-load experiment, all else was the same except for the blood-meal gametocytemia which varied as described in the Results. For each experimental treatment, VS1 or the control compound (PVP) were prepared in PBS and diluted 1∶10 to the final experimental dose with infected blood.

### Recombinant PvCTRP and PvWARP expression

Full-length PvWARP excluding the signal peptide (nt 88–867) and a fragment of PvCTRP containing the first vWA domain (nt 79–921) were PCR-amplified from genomic DNA of the Salvador I strain with a C-terminal 6×His tag appended to the reverse primer. Fragments were cloned into the EcoRV sites of the vector pEU-E01-MCS (Cell Free Sciences, Matsuyama, Japan). The PvCTRP-vWA1 and PvWARP were expressed in the wheat germ cell-free expression system (Cell Free Sciences, Matsuyama, Japan) as described [17] and purified using Ni-affinity chromatography.

### ELISA-based binding and competition assays

Biotinylated VS1-NH_2_ in a volume of 100 µl (10 µg/ml) was applied to each well of a streptavidin-coated (2 µg/ml) 96-well microtiter plate that had been blocked with PBS, 1% BSA (Thermo Pierce) and incubated for two hours at RT. During VS1 incubation, 6×His-tagged recombinant PvWARP and PvCTRP (5 µg/ml) were each mixed separately with heparin and CSA (100 µg/ml) in blocking buffer and incubated for 2 hr at RT. The microtiter plate was subsequently washed three times with PBS to remove excess VS1, and then 100 µl of 6×His-tagged recombinant PvWARP or PvCTRP alone (10 or 5 µg/ml) or of the recombinant protein-GAG mixture was added to each well, with the exception of the no-protein and irrelevant-protein controls. The latter received a 6×His-tagged recombinant glycosyltransferase from *An. gambiae*. Binding and/or competition with VS1 was allowed to proceed for 2 hr at RT. Following three washes with PBS, anti-His MAb (Sigma) was added to each well and incubated for 1 hr at RT. After three washes with PBS + Tween-20 (0.05%), anti-mouse secondary antibodies conjugated to HRP were added and incubated for 1 hr at RT. Following another wash step, TMB ELISA substrate (Pierce) was used for detection. VS1 binding was quantified by measuring the OD at 450 nm with a SPECTRA MAX PLUS microplate reader (Molecular Devices).

### Immunofluorescence microscopy assays

Ookinete samples were fixed with 4% paraformaldehyde and prepared for immunofluorescence microscopy by washing three times with PBS containing 0.1 M glycine (rinsing buffer). To permeabilize samples the parasites were incubated with rinsing buffer containing 0.2% Triton X-100 for 10 min and then washed as before. After the washes, samples were incubated in rinsing buffer for 30 min and blocked with PBS containing 0.05 mM glycine, 0.2% fish skin gelatin and 0.05% sodium azide for 2 hr. The samples were then incubated with biotinylated-VS1 and anti-Pbs21 (*P. berghei*) or anti-Pfs28 (*P. falciparum*) for 1 hr at RT or overnight at 4°C. Cells were washed as before and incubated with Streptavidin, DyLight 488 conjugated (Thermo), and Goat Anti-Rabbit IgG (H+L), DyLight 594 conjugated, for 1 hr at RT. Following incubation, the cells were washed three times with rinsing buffer, resuspended in PBS, spotted on slides and allowed to air dry. ProLong Gold antifade reagent with DAPI (Invitrogen) was added prior to the coverslip and slides were incubated for 24 hr at RT protected from light. Samples were examined with SPOT software using a Nikon Upright E800 microscope.

### Homology models

Homology modeling of the CTRP vWA domain was performed using SWISS-MODEL in two different modes of operation [18]–[21]. In the full-automated mode, the *Toxoplasma gondii* micronemal protein 2 I domain (2XGG, chain B, residues 75–212) was selected as the optimal template to calculate the CTRP model (residues 1–148) and is based on 22% sequence identity. In the template identification mode and using the InterPro Domain Scan method [22], the von Willebrand factor A1 domain (1AUQ, chain A, residues 1276–1463) was selected as the optimal target template (residues 1–193). The model quality was assessed using the QMEAN server [23] and the Z-score.

### Statistical methods

To determine significance between treatment and control groups in the feeding assays, the nonparametric Mann-Whitney U test was used due to the non-normal distributions typical of oocyst counts. For the *in vitro* assays, the test was performed comparing the distribution of oocyst counts per midgut for each treatment group to that of the PVP control, followed by a Bonferroni correction of *z-*scores to adjust for multiple tests. For the *in vivo* assays, the test was performed comparing oocyst counts per midgut between mosquitoes fed on *P. berghei*-infected mice pre- and post-injection with VS1, PVP, or PBS alone.

### Ethics statement

All experimental studies using vertebrate animals (mice) were performed in accordance with Johns Hopkins University (JHU) ACUC (Animal Welfare Assurance #A3272-1) regulations. The Animal Protocol (#MO12H232) used for these studies was reviewed and approved by the JHU ACUC and are in compliance with the United States Animal Welfare Act regulations and Public Health Service (PHS) Policy. No human subject research was performed during this study.

## Results

### Small molecule inhibitors exhibit *Plasmodium falciparum* transmission-blocking activity

Our primary goal was to design synthetic polymers that can mimic sulfated CS-GAGs that have been shown to bind ookinetes (Figure 1A, Figure S1A). However, the likelihood of CS-GAGs, with C4S and C6S sulfation on the midgut microvillar surface was challenged [16]. Using capillary electrophoresis with laser-induced fluorescence detection, we confirmed the presence of both C4S and C6S chondroitin GAGs on *An. gambiae* midgut brush border microvilli vesicles (Figure S1A, Text S1). Based on these data, we hypothesized that polymers with high sulfation densities would be appropriate for our study. As such, two polysulfonated polymers were generated by polymerization of vinyl-sulfonic acid (VS1) and copolymerization of vinyl-sulfonic acid with 1-vinyl-2-pyrrolidone (VS2-PVP). The sulfate groups on the polysulfonated polymers are anionic at physiologic pH and would presumably bind to ookinete proteins that would naturally bind to GAGs on the midgut surface. Note that it was previously shown that the blocking phenomenon is predicted to occur at the apical midgut surface, and that basal lamina GAGs do not influence the ultimate read-out of these T-B studies, which is oocyst prevalence and intensity measurements at 8 or 10 days post-blood feeding, for *P. falciparum* and *P. berghei*, respectively [5].

Initial data from malaria T-B studies indicated that VS1 was non-toxic and well tolerated by both mice and mosquitoes. Although VS2-PVP was tolerated by mice, mosquitoes that ingested the compound had poor survivorship in multiple experiments within 24 hrs following blood feeding. This low survivorship prevented a proper comparison of infection intensity between midguts dissected from pre- and post-injection mosquitoes; and consequently, this compound was not pursued further. In preliminary *in vitro* standard membrane feeding assays (SMFAs), VS1 demonstrated 98.5% and 92.3% inhibition of *P. falciparum* oocyst development in *An. gambiae* and *An. stephensi*, respectively. *In vivo* direct feeding assays (DFA) with mice infected with *P. berghei* demonstrated a somewhat lower effect of the compounds on parasite development in *An. stephensi*, with VS1 reducing oocyst intensity relative to controls by 77%. These preliminary data from both *in vitro* and *in vivo* malaria models demonstrated the potential for VS1 to act as a potent T-B compound. Moreover, to exclude the possibility that micro- and macrogamete fertilization events could be adversely affected by VS1, we tested the effect of the compound on male microgamete exflagellation and noted that the number of exflagellation centers were unaffected (data not shown). We therefore pushed this small molecule forward as the lead compound for further testing, which included (i) assays to determine if T-B activity varies according to polymer length, (ii) dose-ranging assays to estimate the IC_50_ of VS1, (iii) ELISA-based binding and competition assays using a candidate gene approach, specifically recombinant versions of the ookinete micronemal proteins CTRP and WARP, and (iv) immunofluorescence microscopy to confirm binding of VS1 to wild-type ookinetes from both *P. falciparum* and *P. berghei*, as well as ookinetes from knockout lines of *P. berghei*.

### VS1 demonstrates potent transmission-blocking activity across ranges of polymer length, parasite load, and polymer concentration

**Figure 2:**
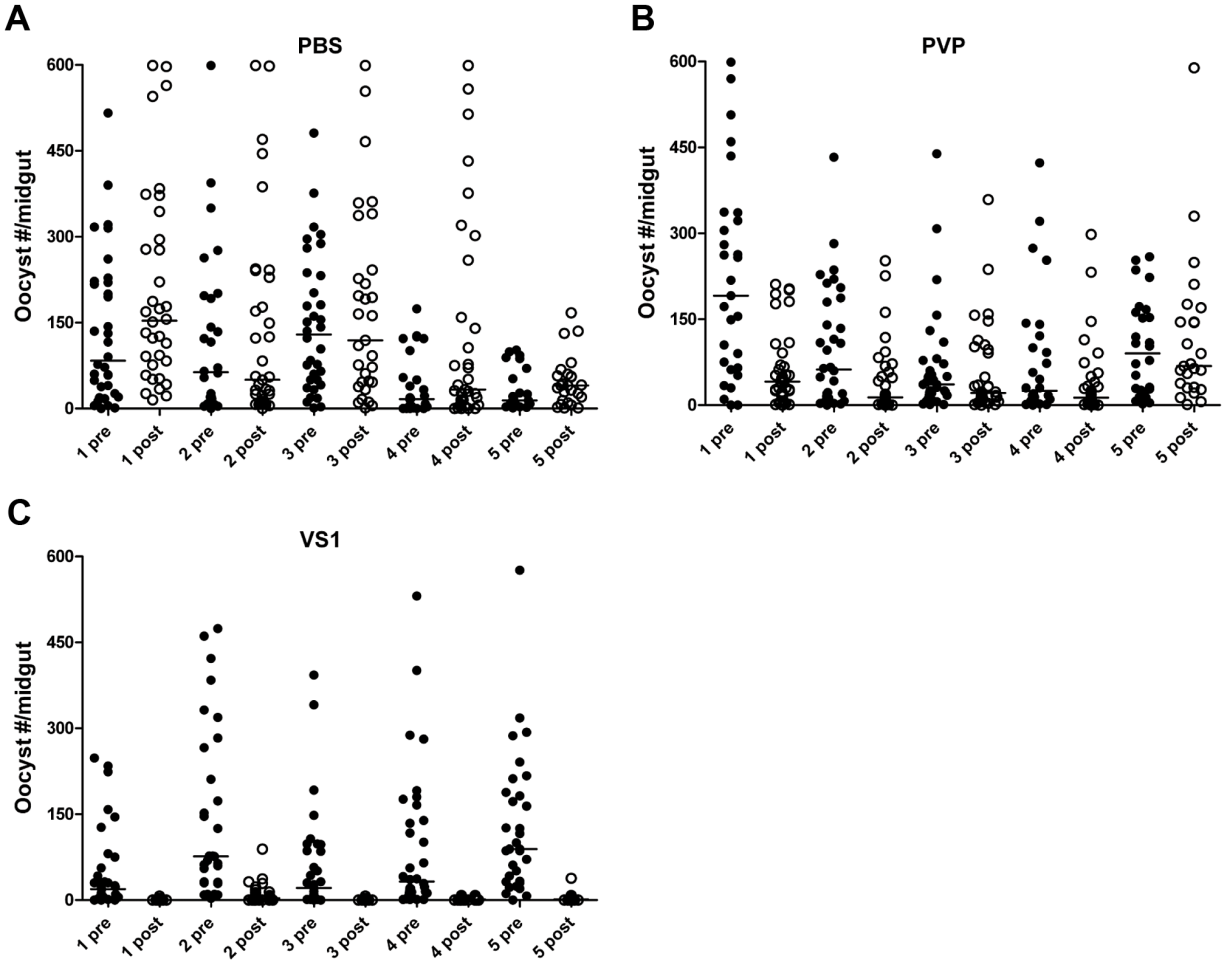
VS1 exhibits malaria transmission-blocking activity in an *in vivo* system. Panels (**A–C**) show midgut oocyst intensities from a representative experiment in which *An. stephensi* mosquitoes were fed on mice infected with *Plasmodium berghei* pre- and post-injection with VS1-3,000. Each panel represents a different treatment group (A, PBS; B, PVP; C, VS1-3,000) and the X-axis of each is arranged by parasitized mouse (labeled 1–5) pre- and post-injection with VS1.

To assess the influence of polymer length, the product following VS1 synthesis was fractionated into three molecular-weight categories by size exclusion chromatography, VS1-10,000; VS1-3,000; and VS1-1,000 (Figure 1A). Based on preliminary dose ranging experiments, each new compound was then tested at a concentration of 250 µg/ml using both *in vitro* (SMFA) and *in vivo* (DFA) malaria models. Polyvinylpyrrolidone, a non-sulfated, neutrally charged control (PVP), which represents the unsulfated VS1 backbone, was used as a control. In SMFAs the three VS1 compounds were tested in parallel against *P. falciparum* in two replicate experiments using two different vector species, *An. gambiae* and *An. stephensi*. In all four experiments, each of the VS1 compounds significantly reduced oocyst intensity (Figure 1B–E). VS1-3,000 consistently performed the best; inhibiting oocyst development by 86.0%–99.0% in *An. gambiae* (Figure 1 B, C) and 88.0%–93.5% in *An. stephensi* (Figure 1 D, E). Experiments with the *in vivo* system yielded similar results, as all three VS1 compounds strongly inhibited *P. berghei* oocyst development in *An. stephensi* (Table 1, Figure 2, Table S1). Results from two replicate experiments per compound showed that all six VS1 treatment groups experienced a highly significant reduction in median oocyst intensity when comparing mosquitoes fed on pre-injection mice with those fed on mice injected with VS1 (Table 1, Table S1). In five of these treatment groups, oocyst development was inhibited by >90%. VS1-3,000 had the strongest effect, demonstrating ≥98.0% inhibition on average in both experiments (Table 1, Table S1B). Conversely, pre- and post-injection comparisons of the oocyst burden in mosquitoes fed on mice from PBS and PVP groups demonstrated no consistent effect of either the VS1 carrier or the unsulfated polymer (Table 1, Table S1). To confirm that VS1 was available to mosquitoes in blood meals, and hence the likely cause of T-B activity, the presence of VS1 in the mouse bloodstream following injection was confirmed by HPLC (Figure S2, Text S1).

**Table 1:** Summary of the results from two replicate direct feeding assays (DFA) for each VS1 compound using *Anopheles stephensi* that fed on *Plasmodium berghei* ANKA 2.34 -infected mice pre- and post-injection with either PBS (carrier-only control), PVP (non-sulfated polymer control), or VS1.

VS1 MW (replicate)	PBS (Pre)	PBS (Post)	% Inhibition	*P*-value	PVP (Pre)	PVP (Post)	% Inhibition	*P*-value	VS1 (Pre)	VS1 (Post)	% Inhibition	*P*-value
**10,000 (1)**	48.1	80.1	−35.2	NS	82.5	47.9	33.7	NS	90.0	3.3	91.1	<0.0001
	(25.34)	(59.68)	(50.81)		(23.95)	(10.28)	(9.58)		(37.33)	(1.65)	(5.55)	
**10,000 (2)**	46.0	24.8	47.3	0.0008	16.3	16.4	−51.4	NS	2.0	0.5	53.3	<0.0001
	(16.84)	(11.24)	(13.12)		(6.20)	(7.04)	(80.41)		(0.37)	(0.26)	(15.92)	
**3,000 (1)**	53.5	30.8	40.4	0.0012	17.9	12.4	−12.6	NS	27.0	0.3	98.8	<0.0001
	(22.83)	(13.23)	(3.81)		(10.68)	(5.68)	(61.55)		(4.86)	(0.22)	(1.12)	
**3,000 (2)**	55.8	60.5	−64.2	NS	53.4	28.9	48.2	0.0001	54.8	1.3	98.0	<0.0001
	(24.10)	(17.72)	(43.61)		(13.06)	(11.78)	(9.96)		(14.79)	(0.56)	(0.80)	
**1,000 (1)**	16.8	12.5	23.2	NS	16.5	13.3	−2.6	NS	14.6	1.0	92.1	<0.0001
	(10.30)	(5.75)	(16.66)		(5.95)	(4.87)	(29.05)		(2.93)	(0.00)	(1.76)	
**1,000 (2)**	41.1	107.5	−140.2	NS	68.6	63.5	−7.6	NS	94.6	2.0	97.8	<0.0001
	(4.80)	(16.91)	(131.38)		(40.57)	(37.77)	(46.54)		(13.27)	(0.73)	(0.92)	

For each treatment group, the data are summarized into four columns. In the first two columns, the means of the median number of oocysts per mosquito for 4–5 mice per group are given for pre- and post-injection feedings, respectively, with the standard error for each reported in parentheses below the mean. In the third column, the means of percent Inhibition, calculated as the average of (median_pre_ – median_post_)/median_pre_ for each mouse, are reported for each treatment group along with standard errors in parentheses. In the fourth column, *P-*value results of Mann-Whitney U tests are reported for each set of pre- and post-injection feedings. Only *P*-values that are significant at a Bonferroni-corrected alpha of 0.0028 are given. NS, non-significant.

**Figure 3:**
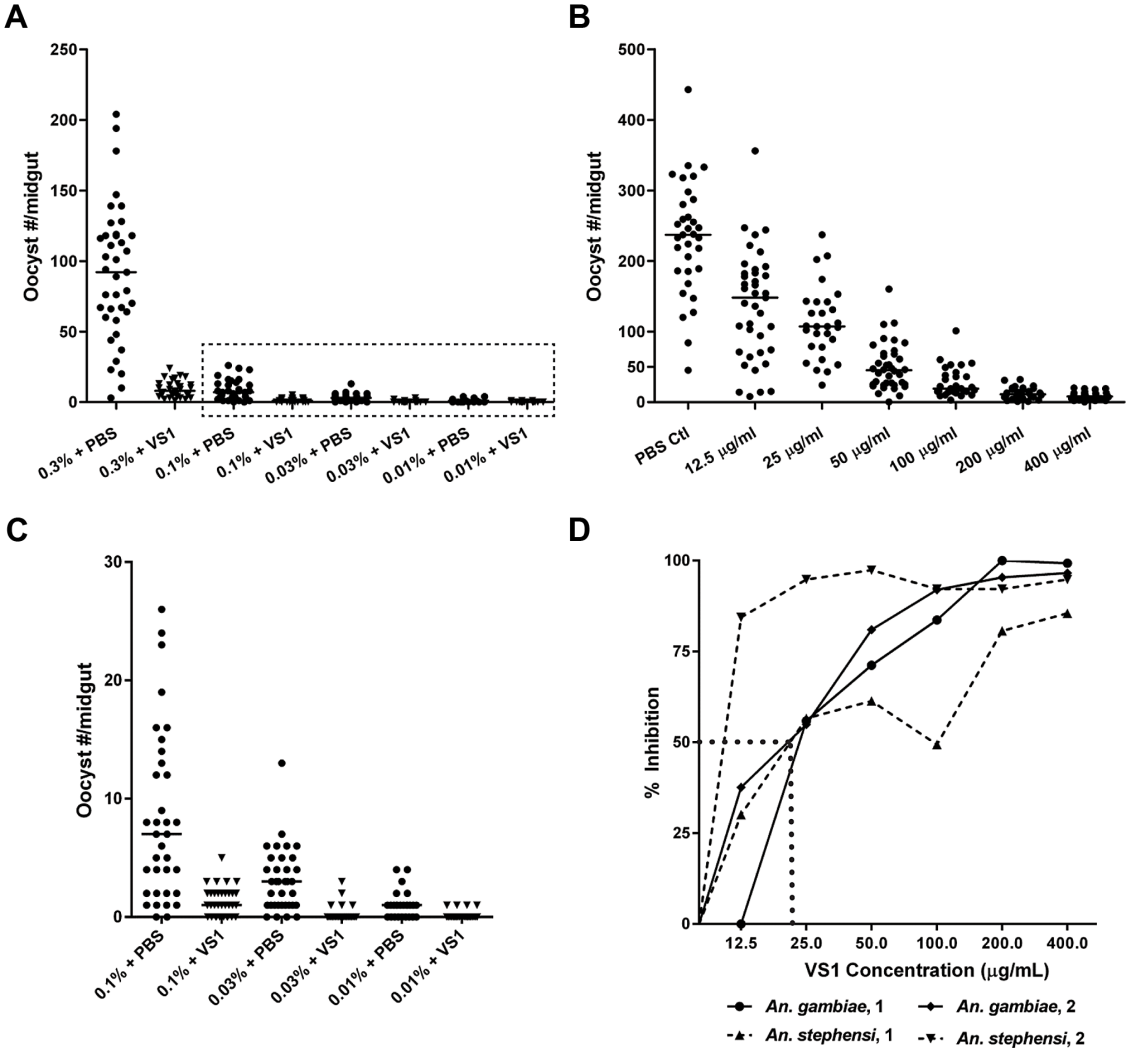
Dose-ranging experiments demonstrate consistent dose-response relationships between gametocytemia, VS1, and percent inhibition of *Plasmodium* infection. (**A**) Results from a gametocyte-dilution SMFA showing the relationship between percent gametocytemia and midgut infection with (X% + VS1) and without (X% + PBS) VS1-3,000. The dashed box denotes the data highlighted in panel C. (**B**) Results from a representative dose-ranging SMFA with *An. gambiae* (replicate 2 in panel D) where mosquitoes were fed *P. falciparum* gametocytes in combination with VS1-3,000 in concentrations ranging from 12.5–400 µg/ml in 2-fold increments. The column headings in the table below are as in Figure 1. (**C**) Results of SMFA experimental treatments in panel A from 0.1% gametocytemia and lower. The data are displayed at a scale that better shows the range of oocyst intensities at lower gametocyte concentrations. (**D**) Relationship between percent inhibition and VS1 concentration from four independent experiments. Each data point represents the percent inhibition of *P. falciparum* infection for a specific concentration of VS1-3,000. Results are shown for two experiments using *An. gambiae* and two using *An. stephensi*. The dashed line approximates the IC_50_.

Due to its consistent T-B activity, VS1-3,000 was selected for use in *P. falciparum* NF54 SMFA experiments to assess the compound's effectiveness across variations in parasite load in the blood meal (gametocytemia) and to estimate the IC_50_ of VS1 in two different anopheline vectors. For the parasite-load experiments, we chose to test VS1 potency at levels of gametocytemia that captured values routinely observed during the conduct of membrane feeding assays in the field [24]–[25]. With the concentration of VS1-3,000 set at 250 µg/ml, a SMFA was performed in which four gametocyte concentrations were tested in the presence and absence of VS1. In this experiment, a day 17 gametocyte culture at 3.0% gametocytemia was pelleted and the packed red blood cells (RBCs) were diluted with uninfected blood to 0.3% and 0.1% gametocytemia. Each of these dilutions was in turn diluted 1∶10 with uninfected blood yielding 4 concentrations that ranged from 0.01%–0.3% gametocytemia (∼800–24,000 stage V gametocytes per µl of blood). The level of gametocytemia commonly used in laboratory-based SMFAs (0.3%) is typically much higher than that found in the field to ensure consistent and robust infections in mosquitoes, allowing more rigorous tests of T-B activity [25]. Though a widely accepted approach, a criticism of the SMFA is that the assay better tests the effects of compounds (or antibodies) on oocyst intensity than prevalence of infection among mosquitoes. Since the ultimate goal is to reduce the latter to zero, we wanted to perform the SMFA over a range of gametocytemias once we established that VS1 consistently reduces the oocyst burden at the usual gametocytemia of 0.3%. In this set of experiments, VS1-3,000 once again demonstrated a potent reduction in oocyst intensity at 0.3% gametocytemia, reducing the median oocyst number per midgut from 92.0 to 8.0 (Figure 3A). However, the prevalence of infection was unchanged between carrier-only and VS1 treatments at this level of gametocytemia. Interestingly, as the gametocytemia was reduced from 0.3%, the effect of VS1 on infection prevalence increased while the reduction in oocyst intensity remained high (Figure 3A, B). In fact, at the two levels of gametocytemia most relevant to the field (i.e., 0.03% and 0.01%), the median oocyst number was reduced to 0 in the VS1 treatments while prevalence was reduced from 89% and 67% in carrier-only treatments to 21% and 13% in VS1 treatments, respectively (Figure 3B, C). In other words, at levels of gametocytemia where untreated mosquitoes averaged fewer than 5 oocysts per midgut and where most mosquitoes were infected, VS1 treatment reduced infection prevalence 4–5 fold and infection intensity by 10 fold. Under these conditions, most VS1-treated mosquitoes were uninfected, while the few that were tended to have a single oocyst.

In a set of two dose-ranging experiments with *An. gambiae* and two with *An. stephensi*, VS1-3,000 was fed to mosquitoes in infectious blood meals using serially diluted concentrations from 400 µg/ml to 12.5 µg/ml. The four experiments revealed a consistent pattern of percent inhibition characterized by a linear increase from little to no inhibition at 12.5 µg/ml to approximately 80% at 100 µg/ml and then a plateau >90% at concentrations >200 µg/ml (Figure 3D). From these data the IC_50_ of VS1-3,000 was approximated to be 25 µg/ml.

### VS1 shows binding affinity to *Plasmodium* ookinetes

**Figure 4:**
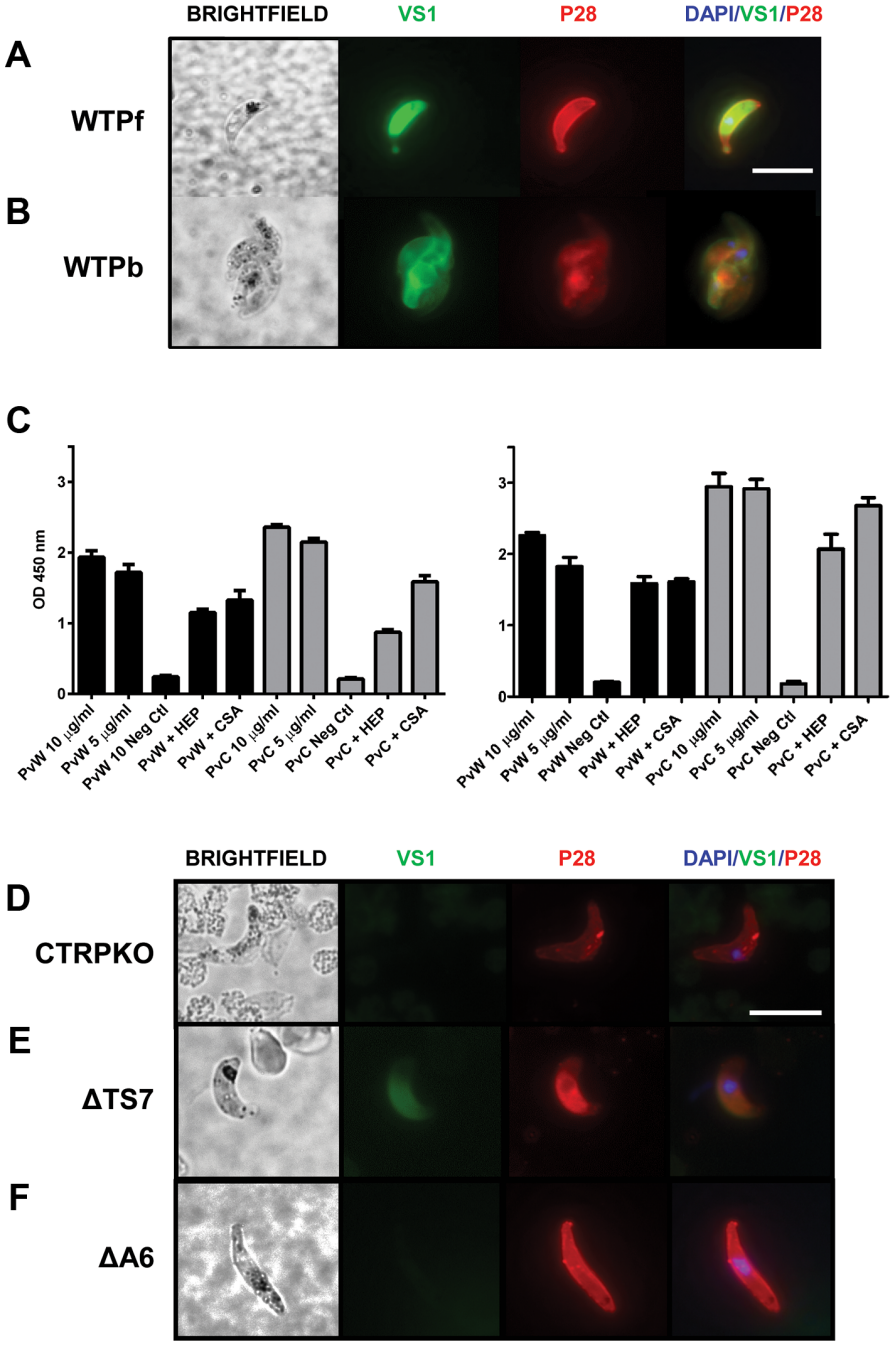
VS1 binds to critical *Plasmodium* micronemal proteins. Immunofluorescence microscopy images of VS1 staining patterns of permeabilized wild type ookinetes from (**A**) *P. falciparum* (WT_Pf_) and (**B**) *P. berghei* (WT_Pb_). Each row of images depicts brightfield, followed by staining with VS1 (green), P28 (red), and the merged image of VS1, P28, and DAPI nuclear staining (blue). Note that different antibodies were used for each *Plasmodium* species to stain orthologous surface markers, α-P28 for *P. falciparum* and α-Pbs21 for *P. berghei*. Size bar = 10 µm. (**C**) Binding assays with recombinant *P. vivax* CTRP and WARP (Figure S5) demonstrate that biotinylated VS1 is recognized by PvWARP and the first vWA domain of PvCTRP. Two representative experiments are shown with assays performed in triplicate. Black bars represent PvWARP (PvW) and gray bars represent PvCTRP (PvC). Each protein, at two concentrations (10 and 5 µg/ml), was allowed to bind with biotinylated VS1 immobilized to a microplate, followed by detection using an anti-HIS MAb (Sigma). In addition, competitive binding assays were performed by incubating the recombinant proteins with heparin (HEP) and chondroitin sulfate A (CS-A) prior to incubation with VS1 and detection of PvWARP and PvCTRP binding to the VS1-coated plate as above. A no-protein control was included in each ELISA assay and used as the background subtraction value. Error bars represent +/−1 standard deviation. (**D–F**) Immunofluorescence microscopy images demonstrating selective VS1 binding to ookinetes that were generated *in vitro* from *P. berghei* (**D**) CTRP_KO_, (**E**) ΔTS_7_, and (**F**) ΔA_6_ transgenic lines. VS1 staining of ΔTS_7_ but not CTRP_KO_ or ΔA_6_ ookinetes suggest that the vWA domain of CTRP is the primary binding ligand of VS1, and that staining is specific to the localized expression of CTRP in micronemes. Each row of images depicts brightfield, followed by staining with VS1 (green), P28 (red), and the merged image of VS1, P28, and DAPI nuclear staining (blue). Size bar = 10 µm.

Since VS1 is a hypothesized structural mimetic of midgut-microvillar sulfated GAGs, we sought to determine whether VS1 can directly bind to *Plasmodium* ookinetes. To this end, we used biotinylated VS1-NH_2_ (Figure 1A) to probe non-permeabilized and permeabilized *P. berghei* ookinetes generated *in vitro*, as well as *ex vivo* blood-meal derived *P. falciparum* ookinetes isolated from dissected mosquito midguts. Only permeabilized *Plasmodium* ookinetes showed strong binding affinity to VS1 by immunofluorescence microscopy. The VS1 staining pattern suggested that it is not associated entirely with the ookinete surface since it did not consistently bind to non-permeabilized ookinetes nor did it localize with the abundant ookinete surface marker P28 (also called Pbs21 in *P. berghei*) in either *P. falciparum* (WT_Pf_) or *P. berghei* (WT_Pb_) (Figure 4A, 4B). VS1 binding appears to be centrally and apically localized in the cytoplasm, suggestive of interaction with micronemal proteins since these proteins are not constitutively secreted to the ookinete surface and are stored in the micronemes [10], [26]. In addition to ookinetes, VS1 also bound to *P. falciparum* retorts (i.e., developing ookinetes) (data not shown), and a portion of permeabilized *P. falciparum* and *P. berghei* round cells. These cells are likely to be zygotes or unfertilized macrogametes (Figure S3E–L) since they are stained with P28, a surface marker well described in the literature, which is expressed from macrogametes to early oocyst [27]–[29] ruling out the possibility that these cells are *P. berghei* or *P. falciparum* gametocytes. Furthermore, *P. falciparum* stage IV and V gametocytes are not round but have a distinctive elongated morphology, and these cells did not stain withVS1 in subsequent immunofluorescence experiments targeting gametocytes (Figure S4A–D, Text S1). We cannot rule out an effect of VS1 in macrogametes and/or zygotes because those stages were not the focus of the current research, but any effect in these stages will potentiate the effect of VS1 against transmission of *Plasmodium*. We evaluated the effect of VS1 in the ability of microgametes to exflagellate in *P. berghei* and found that VS1 had no inhibitory effect on exflagellation between pre- and post-injection mice (Table S2). Furthermore, dissection of mosquitoes 24 hours after feeding with either PBS or VS1 showed presence of ookinetes in the blood meal (data not shown). Nevertheless, more studies are needed to more thoroughly assess the effect of VS1 on macrogametogenesis, fertilization, and ookinete development. Because we are working with a T-B compound, targeting more than one stage of the parasite in the mosquito gut would strengthen the outcome of our final goal, completely blocking transmission of malaria.

### VS1 binds to recombinant CTRP and WARP proteins

We used a candidate gene approach to identify potential targets or binding ligand(s) of VS1 among the repertoire of *Plasmodium* ookinete micronemal proteins [30], focusing particularly on those with established roles in midgut attachment or invasion. The literature indicates that two such proteins, CTRP and WARP, bind to sulfated GAGs [10]; and although both are in the apicomplexan TRAP/MIC2 family of proteins, their domain architectures are quite different [30]. WARP is an approximately 40 KDa protein with a signal peptide and a single vWA domain, and we hypothesize that based on the published data WARP is secreted from the ookinete microneme and can thus work as an extracellular adaptor protein, potentially bridging the parasite surface (or surface molecules) with midgut apical membrane ligands. The much larger CTRP is approximately 230 KDa and contains a signal peptide followed by six contiguous vWA domains, seven contiguous thrombospondin (TS) domains, a transmembrane domain, and a short acidic cytoplasmic domain at the C-terminus that interacts with the motility actomyosin machinery [30]. The first four vWA domains of CTRP are more similar to one another than to vWA domains 5 and 6 when comparing six species of *Plasmodium*. Interestingly, a phylogenetic analysis of the vWA domains from TRAP, CTRP, and WARP among these species shows that WARP and CTRP form a single clade and that the vWA domain of WARP most recently shared a common ancestor with the fifth vWA domain of CTRP, suggesting that WARP evolved from CTRP [30]. We emphasize that the domain architectures and amino acid sequences of these two proteins are highly conserved across Plasmodia [10], [30] and argue that VS1's potency against both rodent and human malaria, as well as its ookinete staining pattern as reported here, suggests that VS1's binding partner(s) is likewise highly conserved across Plasmodia. Thus given the above and the aim of identifying the mechanism of action for VS1, we sought to investigate the binding activity of *Plasmodium vivax* CTRP and WARP to VS1, following the argument that VS1 should bind to these two molecules. We produced soluble recombinant WARP and the first vWA domain of CTRP from *P. vivax* using a cell-free wheat germ system (Figure S5) and evaluated binding affinity via ELISA. As expected, both recombinant PvCTRP and PvWARP bound to VS1 in a dose-dependent manner (Figure 4C). To better delineate binding specificity, competition assays with heparin and chondroitin sulfate A (CSA) were performed. If VS1 binds primarily to the putative GAG-binding sites on the vWA domains of CTRP and WARP, then we would expect that heparin, and perhaps to a lesser extent CSA, at a concentration of 100 µg/ml should completely inhibit binding. However, we observed that both heparin and CSA only partially inhibited VS1 binding to PvCTRP and PvWARP (Figure 4C), suggesting that VS1 binds to additional sites not used by heparin on either recombinant protein or that it binds to them with greater affinity.

### VS1 does not bind to CTRP- or vWA domain knockout lines of *P. berghei*

The ELISA results demonstrated that VS1 binds to recombinant WARP and the first vWA domain of CTRP *in vitro*, so to test binding *in vivo* we obtained three lines of *P. berghei* in which CTRP had been either completely [9], [15] or partially knocked out [15] and compared VS1 staining patterns by immunofluorescence microscopy. One of the partial knockouts, a line known as ΔA_6_, expresses CTRP that is missing all six of the vWA domains but contains all seven of the thrombospondin domains. Conversely, CTRP expressed by the other partial knockout line, ΔTS_7_, includes the six vWA domains but lacks any of the thrombospondin domains. Since the recombinant CTRP protein used in the ELISA consisted only of the first vWA domain, we predicted *a priori* that the VS1 binding pattern to ΔTS_7_ ookinetes would be similar to wild type ookinetes, while the VS1 signal in both the CTRP knockout (CTRP_KO_) and ΔA_6_ lines would be diminished. However, if VS1 also binds to any of the TS domains *in vivo*, the VS1 signal would be much lower in the CTRP_KO_ than in either of the partial knockouts. A caveat to this approach is that if VS1 also binds to WARP *in vivo*, we would expect some portion of the VS1 signal observed in wild type ookinetes to be shared among all of the knockout lines.

Immunofluorescence microscopy images from CTRP_KO_ (Figure 4D) indicate that VS1 binds to CTRP in permeabilized ookinetes. Furthermore, comparisons of staining patterns from the partial knockouts strongly suggest that VS1 binding involves vWA domains but not the TS domains (Figure 4E, F). Furthermore, the apparent loss of VS1 signal in both the CTRP_KO_ and ΔA_6_ lines also suggests that VS1 localizes to the micronemes (as suggested by Figure 4B) and that CTRP is the primary micronemal target of VS1and not WARP (Figure 4D, F). These data further reconcile the observed cytoplasmic staining of putative zygotes/macrogametes (Figure S3) with the previously reported staining of round forms with CTRP antisera [11]. Without a WARP knockout line we cannot rule out that binding to WARP may occur *in vivo* or that some of the T-B activity we observed is due to such an interaction. It should be noted that WARP expression/secretion is not well understood and may be temporally regulated or even midgut contact-dependent for different *Plasmodium* species. Thus, *in vitro* generated *P. berghei* ookinetes or *P. falciparum* ookinetes isolated from the blood-meal bolus may not express detectable levels of WARP. However, the CTRP_KO_ and ΔA_6_ microscopy data are persuasive and we note that VS1 binding by ELISA is consistently stronger for the first vWA domain of CTRP than for WARP. Moreover, the phylogenetic relationship among CTRP and WARP vWA domains [30] in combination with the ELISA and immunofluorescence microscopy data reported here, suggest that VS1 primarily targets the first four vWA domains of CTRP. The divergence of vWA domains 5 and 6 and their evolutionary relationships with WARP suggest that these domains bind VS1 secondarily or not at all.

### Homology model predicts GAG-binding sites in CTRP vWA domain

**Figure 5:**
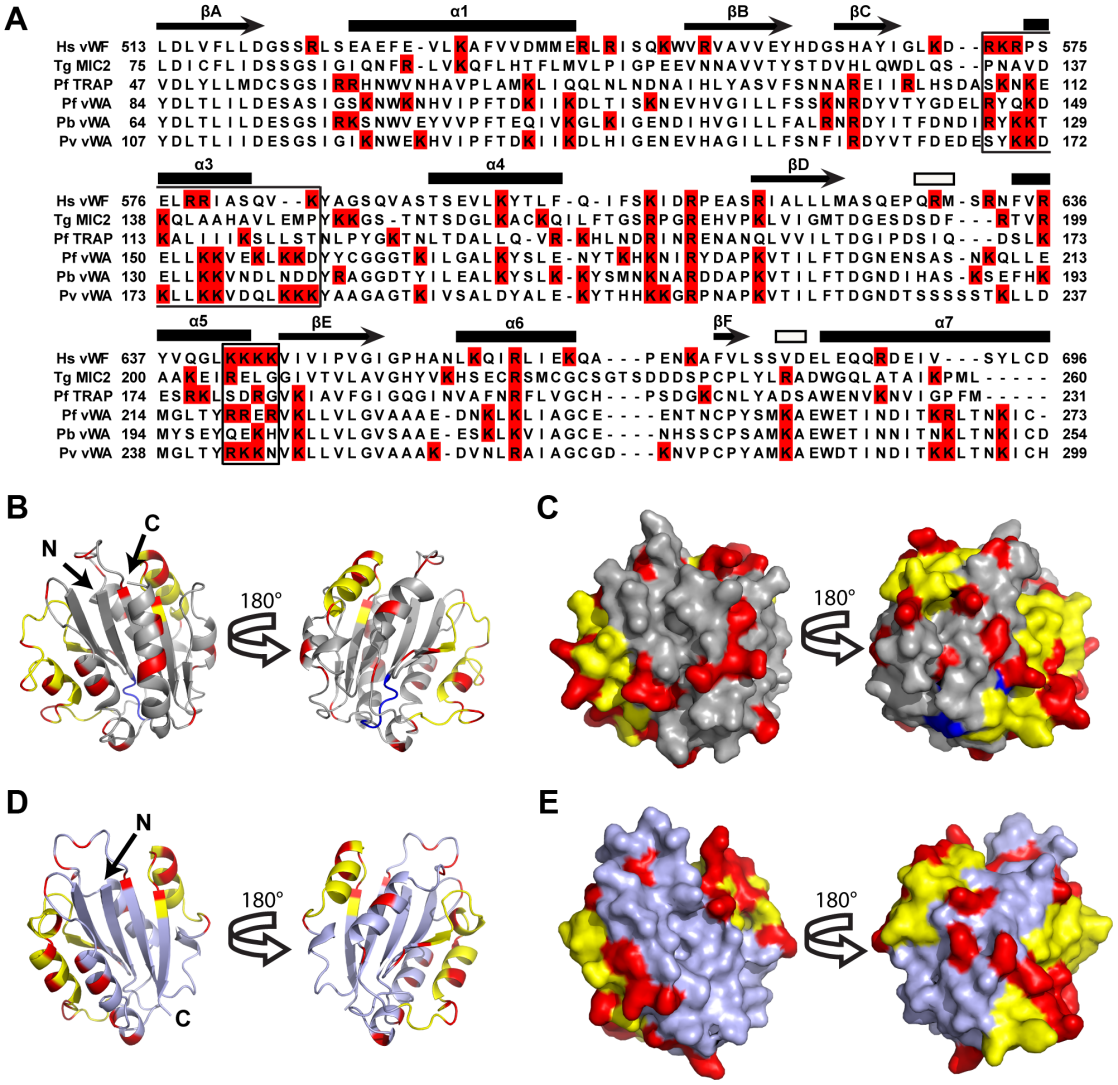
A Homology model of *Plasmodium falciparum* CTRP provides insight into the potential VS1 binding motifs. (**A**) Amino acid alignment of CTRP vWA domain 1 from *P. falciparum* (PfCTRP 1), *P. berghei* (PbCTRP 1), and *P. vivax* (PvCTRP 1) with vWA domains from *P. falciparum* thrombospondin-related anonymous protein (PfTRAP), *T. gondii* micronemal protein 2 (TgMIC2), and human von Willebrand Factor (Hs vWF A1). Arginine and Lysine residues are highlighted in red and motifs involved in heparin-binding in Hs vWF A1 are boxed. Secondary structural elements for Hs vWFA1 are provided above each row of amino acids as described [32]. Arrows represent beta strands (labeled A–F), closed rectangles represent alpha helices (labeled 1–7; α2 intentionally missing due to lack of equivalent helix relative to other human vWA domains), and open rectangles represent 3–10 helices. (**B–C**) Homology model (light gray) of *P. falciparum* CTRP (Model 1) based on the crystal structure of the human vWF A1 domain (PDB: 1AUQ) and the same Model rotated 180°. In (B) the homology model is presented as a ribbon diagram, while a space-filling model is shown in (C). (**D–E**) Homology model (steel gray) of *P. falciparum* CTRP based on the *Toxoplasma* MIC2 structure (PDB: 2XGG) and the same model rotated 180°. In (D) the homology model is presented as a ribbon diagram, while a space-filling model is shown in (E). In (B–E) yellow residues indicate the predicted heparin-binding domains for Models 1 and 2 that have been described for the vWF A1 domain and indicated as boxed areas in the sequence alignment in panel (A). Red residues indicate the positively charged Arg and Lys moieties present outside of the predicted heparin-binding domains (in yellow) for Models 1 and 2. Blue residues correspond to the putative MIDAS metal-ion binding motif [DXSXR in vWF A1, DXSXS in the apicomplexan vWA domains included in panel (A)].

In the absence of a CTRP crystal structure, we used homology modeling to predict heparin-binding sites on CTRP (Figure 5A–E). The quality of the models was assessed with QMEAN; and models 1 (PDB: 1AUQ, Figure 5B, C) and 2 (PDB: 2XGG, Figure 5 D, E) had Q_mean_ scores of 0.572 (Z-score = −2.892) and 0.584 (Z-score = −2.314), respectively, indicating comparable model reliability. Model 1 is based on the human von Willebrand factor A1 domain, while Model 2 is based on the vWA-integrin like domain from the *Toxoplasma gondii* MIC2 protein. The structure of the former has been extensively studied due to its essential role in platelet adhesion [31]–[33], while the latter is a well-described adhesin involved in host-cell invasion [34]. Despite the selection of markedly different templates, both with low sequence identity to CTRP (<25%), both models had the same overall α/β Rossmann fold. The positively charged residues adopted similar patterns between the two models (Figure 5 B–E), which were also in general agreement with the predicted heparin-binding domains on vWFA1 (Figure 5A). A superposition of Model 1 with vWFA1 (1AUQ) (Figure S6A–B) suggests that the two models predominantly differed in their overall length and the conformation of the loops connecting the beta sheet core and flanking alpha helices. A superposition of Models 1 and 2 also demonstrated a difference in overall length as well as the presence of a large alpha helix in Model 1 that is absent in Model 2 (Figure S6C–D). Furthermore, a number of basic residues fall well outside the predicted vWFA1 heparin-binding regions in CTRP (Figures 5A–E), which may represent an extended electropositive surface and additional binding sites for sulfated polymers such as heparin and VS1.

## Discussion

Host-cell GAGs have been shown to be important mediators of *Plasmodium* development in its two hosts, including merozoite invasion of RBCs [35]–[36], infected RBC sequestration to placenta [37], ookinete invasion of the midgut [5], and sporozoite invasion of mosquito salivary glands [38] and vertebrate hepatocytes [39]. Here we tested a strategy that exploits this feature of *Plasmodium* biology and demonstrated that VS1, a putative GAG-mimetic, reduced midgut oocyst development by as much as 99% in mosquitoes fed with *P. falciparum* or *Plasmodium berghei*. Through direct-binding assays, we observed that VS1 bound to two ookinete micronemal proteins necessary for midgut invasion, each containing at least one vWA domain: (i) CTRP and (ii) WARP. By immunofluorescence microscopy, we observed that VS1 stains permeabilized *P. falciparum* and *P. berghei* ookinetes but does not stain *P. berghei* CTRP knockouts or transgenic parasites lacking the vWA domains of CTRP while retaining the thrombospondin repeat region. Finally, we used structural homology models of the first vWA domain of CTRP to identify residues likely involved in binding GAGs, as well as the VS1 compound. Based on these data, our working model for the mechanism underlying VS1's T-B activity is that it binds to CTRP once the protein is secreted from the micronemes of ookinetes prior to midgut attachment and invasion. CTRP is essential for gliding motility [9], [15] and contains six vWA domains, which commonly play roles in cell adhesion to GAGs [8], [10], [30]–[34]. Our data suggest that VS1 either interrupts the gliding process on the midgut apical microvillar surface or coats the surface of the ookinete through its interaction with CTRP, thus preventing attachment to ligands (e.g., chondroitin sulfate [5]) on the apical surface of the midgut epithelium. Nevertheless, due to observed binding of VS1 to permeabilized round cells, we cannot rule out that VS1 may potentially have an added benefit and affect additional parasite stages found in the blood meal, particularly macrogametes and/or zygotes. Further studies into these beneficial side effects are necessary.

When designing the GAG-mimetic strategy, data from the literature suggested that sulfation density per disaccharide unit and the manner of presentation (i.e., how the underlying structure of the sugar scaffold influences the 3D projection of sulfated moieties) are critical factors in inhibiting pathogen-GAG interactions. Boyle et al. [35], for example, found that heparin and the *E. coli-*derived K5 polysaccharide inhibits merozoite entry into RBCs and that variations in the average number of sulfate groups/saccharide unit for K5, which consists of glucoronate as opposed to iduronate, exhibited different inhibitory effects against merozoites, with sulfate densities >3/disaccharide producing the most potent IC_50_ estimates. Therefore, we sought to determine the minimal T-B polymer length of VS1 with the hope of minimizing the likelihood of diverse structural conformations that can occur with longer polymers, which could in turn affect the presentation/projection of anionic moieties. Although we were able to demonstrate that VS1-3,000 was the most effective polymer, we cannot, however, predict its structure. VS1-3,000 is unlikely to remain linear in solution or in the midgut after blood feeding. With this caveat in mind, we suspect that binding to the recombinant or native CTRP and WARP molecules may engender a specific VS1 conformation. Regardless, we expect that VS1 binding is largely due to the predicted GAG-binding motifs on the vWA domain(s) of CTRP and WARP [10]. However, the concentration of heparin used in our studies, which would otherwise result in the near complete inhibition of high affinity protein-GAG interactions [40]–[42] only reduced VS1 binding by ∼25%. It should be noted, however, that cases exist in the literature where soluble heparin cannot completely outcompete vWA domain-GAG interactions. For example, heparin-BSA binding to the vWA domain of PfTRAP can be competed between 45–66% using 50 µg/ml of soluble heparin and that a 10-fold increase in heparin concentration reduced binding to 9–27% of control [43]. Even more striking is a report that neither a 50-, 100-, or 1,000-fold molar excess of soluble heparin could completely inhibit binding between the PfTRAP vWA domain and the surface of HepG2 cells, which was thought to be GAG mediated [44]. In this set of experiments, each concentration reduced binding by approximately 15%, 55%, and 70%, respectively, suggesting that the PfTRAP vWA domain utilizes both GAG and a non-GAG binding sites. In combination, these data suggest that each recombinant protein in our study has either stronger binding affinity for VS1 than for either heparin or CSA, that the predicted GAG-binding regions do not completely explain the interactions of VS1 with PvCTRP or PvWARP, or more likely, a combination of these two scenarios. Tertiary structures of mammalian heparin-binding proteins have also been shown to enhance affinity and specificity [41]. We cannot rule out the possibility of cryptic GAG-binding sites on CTRP and WARP that provide cooperative binding to VS1, as suggested at least in part by potential basic residue patches identified on two homology models of the first vWA domain of CTRP, which appears to be a primary ligand of VS1. In terms of sulfation density and propensity to form various non-linear conformations, VS1 is clearly different from Heparin and natural GAGs. In this context, cooperative binding may be conferred by VS1 “wrapping around” CTRP and interacting with basic residues along different faces of the protein. The presence of potential additional binding sites suggests that CTRP (as opposed to other GAG binding proteins) can be specifically targeted by the next generation of VS1-based chemical mimetics. Clearly, a crystal structure for CTRP is needed to clarify the hypotheses generated by our two models.

To date, the antimalarial pipeline is filled with compounds that act on related biochemical pathways (e.g., folate biosynthesis), which also increase the likelihood of the development of parasite cross-resistance to these “new” compounds. The need to discover drugs that act on unpredicted or uncharacterized biochemical pathways that are completely different from those associated with current antimalarials is paramount [45]. Our approach fits this mold, as it represents a completely novel mechanism of action compared to those associated with the existing, new, and now “rediscovered” list of antimalarials and T-B compounds [7].

Among the various T-B strategies, drugs offer a distinct advantage over vaccines since the efficacy of the compound is dose dependent and human immune-system independent, the latter being a potentially significant issue given that individuals in malaria endemic regions may suffer from malnourishment and concomitant infections by immune-modulating pathogens such as HIV and helminths. Although we have shown that VS1 is a potent T-B molecule, we emphasize that it cannot be used as a drug in its current form. However, we intend to use the data reported here to establish a high-throughput approach for identifying a next-generation “druggable” malaria T-B compound that would inhibit ookinete invasion of the midgut beyond that observed for VS1 (i.e., achieve zero infection prevalence among treated mosquitoes). We recognize, however, that if the next generation compound only replicates the T-B activity reported for VS1 and were used alone in the field, it would unlikely reduce infection prevalence in the mosquito population below the threshold necessary for sustained transmission . Nevertheless, it is widely believed that no anti-malarial intervention on its own will lead to regional elimination and eventual eradication [1], [2]. We envision that in this context, such compounds may be valuable in a range of epidemiologic settings. Potential applications include (i) general use in conjunction with existing artemisinin combination therapies, which we emphasize do not kill stage V gametocytes, to prevent recurrent transmission from the treatment-seeking segment of the population, (ii) use in regions with unstable malaria (e.g., highlands) to curb transmission during epidemics, (iii) use in combination with a T-B vaccine targeting sexual stage parasites to act as a safety net to “mop up” break-through parasites, and (iv) at the end game of the malaria eradication effort, as mass distribution of T-B compounds may offer a cost-effective approach to preventing asymptomatic, gametocytemic individuals, who would not otherwise seek treatment, from infecting anopheline mosquitoes, thus preventing resurrection of epidemic malaria transmission.

## Supporting Information

Figure S1 **Capillary electrophoresis laser-induced fluorescence (CELIF) spectroscopy identifies the C6S moiety on chondroitin sulfate glycosaminoglycans on the apical midgut brush border microvilli vesicles (BBMV) of** ***Anopheles gambiae*** **.** (**A**) Structure of chondroitin sulfate (CS) disaccharide units likely found on the apical surface of midgut epithelial cells in *An. gambiae* and that have been shown to bind directly to *P. falciparum* ookinetes *in vitro* [5]. C6S is present in both CSC and CSE. (**B**) CELIF analysis. Trace A, baseline *An. gambiae* midgut BBMV isolated from 1,500 5–6 day old female, sugar fed *An. gambiae*. Trace B, BBMV + ΔDi6S disaccharide added to the sample. Trace C, BBMV + ΔDi4S and ΔDi6S disaccharides added. Trace D, Chondroitin sulfate C (CSC) control. Red arrows indicate the presence of C4S and C6S modifications to chondroitin sulfate on the midgut surface, as evident from the increased peak intensities following the addition of the disaccharides as compared to the CSC control which has both C4S and C6S modifications.
(TIF)

Figure S2 **HPLC-based quantification of VS1 in mouse blood.** (**A**) Peak absorbance at 210 nm was measured for VS1-biotin standards ranging in concentration of biotin from 25–200 nmol/ml (corresponds to 0.1375–1.1 mg/ml of biotinylated VS1-NH_2_). The table below the graph provides the integrated area under the peak for each standard, as well as those values with the blank subtracted (Area_sub_). The inset graph shows the standard curve generated from the linear relationship of integrated area under the peak and concentration of biotin. (**B**) Peak absorbance at 210 nm was measured for three technical replicates (TR) of VS1-biotin isolated from mouse blood. The table below the graph reports integrated area under the peak for each TR, those values with the blank subtracted (Area_sub_), and the estimated concentration based on the standard curve.
(TIF)

Figure S3 **Additional microscopy images demonstrating VS1 binding to mosquito-stage parasites.** (**A–D**) Brightfield and immunofluorescence microscopy images of VS1 staining patterns of permeabilized wild type ookinetes from *P. berghei*. Each pair of images depicts brightfield (A, C), followed by staining with VS1 (green) merged with DAPI nuclear staining (blue) (B, D). The arrow in panel B highlights greater VS1 staining intensity apically. Size bar = 10 µm. (**E–L**) Microscopy images of VS1 staining patterns of permeabilized round cells from wild type *P. falciparum* (E–H) and *P. berghei* (I–L). Each row of images depicts brightfield (E,I), followed by staining with Pfs25 (red) (F) or P28 (red) (J), VS1 (green) (G, K), and the merged image of VS1, PfS25/P28, and DAPI nuclear staining (blue). Size bar = 10 µm.
(TIF)

Figure S4 ***Plasmodium*** **gametocytes are not stained with biotinylated VS1.** (A–D) Brightfield and fluorescence microscopy images of VS1 staining patterns for non-permeabilized wild type gametocytes from *P. berghei* (A–B) and *P. falciparum* (C–D). Each pair of images depicts brightfield (A, C), followed by staining with VS1 (red) merged with DAPI nuclear staining (blue) (B, D). Black and white arrows denote the location of the gametocytes. Size bar = 10 µm.
(TIF)

Figure S5 **SDS-PAGE gel demonstrating the wheat germ cell free system for soluble expression of His-tagged recombinant** ***Plasmodium vivax*** **CTRP and WARP.** Asterisks denote the sample material used in subsequent assays. M: molecular weight marker. T: Total translation mix. S: Supernatant. P: Pellet. Ft: Flow through. Ne: Non-reducing condition elution. Re: Reducing condition elution. R: Resin. Scale: 6 µl/well. Concentration: PvCTRP (162 µg in 200 µl) and PvWARP (58 µg in 200 µl).
(TIF)

Figure S6 **VS1, a proof of concept molecular mimic of apical midgut chondroitin sulfate glycosaminoglycan binds to** ***Plasmodium*** **Circumsporozoite and Thrombospondin Related Anonymous Protein Related Protein (CTRP).** (**A**) Homology model of *Plasmodium falciparum* CTRP (Model 1) based on the crystal structure of human von Willebrand Factor A1 (vWFA1) domain (PDB: 1AUQ). (**B**) Superposition of the PfCTRP Homology (Model 1, gray) on the vWFA1 crystal structure (1AUQ, blue). Structures/models for (B) and (C) are in same orientation as Figure 5 (B–E). Note that the structure and model are similar, with the exception of a few loops and the N- and C-termini. (**C**) Superposition of Model 1 (gray) and Model 2 (Fig. 5 (D–E), based on *Toxoplasma* MIC2 structure, 2XGG) and the same image rotated 180° in (**D**). The main difference between Model 1 and Model 2 is shaded in red, which is an extra helix modeled in Model 1 but is missing in Model 2.
(TIF)

Table S1 **Data from direct feeding assays (DFAs) summarized in** **Table 1** **using the three compounds tested** ***in vivo*** **with the** ***Plasmodium berghei*** **/mouse system.** Each tab includes data from two replicate experiments for each compound and set of controls, both-pre- and post-injection into *P. berghei*-infected mice. The tabs are labeled as follows: S1A = VS1 10,000; S1B = VS1 3,000; S1C = VS1 1,000. Data provided include the total number of mosquitoes dissected and scored for oocysts after feeding on a given mouse (N), the number of mosquitoes not infected (# Not Infected), the percent prevalence (Prevalence), the median number of oocysts per midgut (Median), the mean number of oocysts per midgut (Mean), and the percent inhibition (% Inhibition) (for post-injection only, calculated as described in Table 1).
(XLSX)

Table S2 **Male gametocyte exflagellation data from** ***Plasmodium berghei*** **ANKA 2.34-infected mice pre- and post-injection with either PBS (carrier-only control), PVP (non-sulfated polymer control), or VS1-3000.** Exflagellation data from groups of 5 mice are presented in three columns per treatment group. The first two columns show the average number of exflagellation centers per 40× field from five fields recorded both pre- and post-injection for individual mice. The third column provides the *P*-value for a comparison at the group level of the average number of exflagellation centers in pre- versus post-injection mice. Note that a different set of mice was used for each treatment (i.e., Mouse 1 is not the same between PBS, PVP, and VS1 treatment groups).
(DOCX)

Text S1 **This file contains the materials and methods corresponding to the supplementary Figure S1 (Capillary Electrophoresis Laser Induced Fluorescence analysis of midgut BBMV Chondroitin sulfate GAGs), Figure S2 (Determination of VS1 concentration in mouse blood), and Figure S4 (** ***Plasmodium*** **gametocytes immunostaining).**
(DOCX)
