# Supplementary figures and images for: A Small Molecule Glycosaminoglycan Mimetic Blocks Plasmodium Invasion of the Mosquito Midgut
Source: PLoS Pathog. 2013 Nov 21;9(11):e1003757. doi: 10.1371/journal.ppat.1003757 (PMC3836724; doi:10.1371/journal.ppat.1003757)

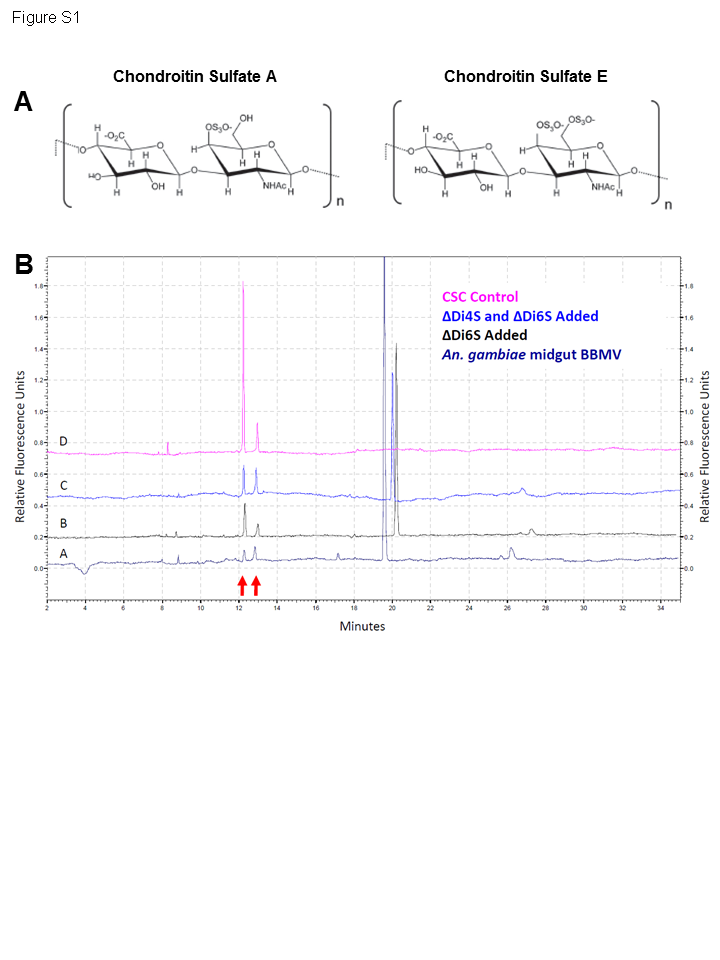

Supplement: Figure S1 — Capillary electrophoresis laser-induced fluorescence (CELIF) spectroscopy identifies the C6S moiety on chondroitin sulfate glycosaminoglycans on the apical midgut brush border microvilli vesicles (BBMV) of Anopheles gambiae . (A) Structure of chondroitin sulfate (CS) disaccharide units likely found on the apical surface of midgut epithelial cells in An. gambiae and that have been shown to bind directly to P. falciparum ookinetes in vitro [5]. C6S is present in both CSC and CSE. (B) CELIF analysis. Trace A, baseline An. gambiae midgut BBMV isolated from 1,500 5–6 day old female, sugar fed An. gambiae. Trace B, BBMV + ΔDi6S disaccharide added to the sample. Trace C, BBMV + ΔDi4S and ΔDi6S disaccharides added. Trace D, Chondroitin sulfate C (CSC) control. Red arrows indicate the presence of C4S and C6S modifications to chondroitin sulfate on the midgut surface, as evident from the increased peak intensities following the addition of the disaccharides as compared to the CSC control which has both C4S and C6S modifications. (TIF) [file ppat.1003757.s001.tif]

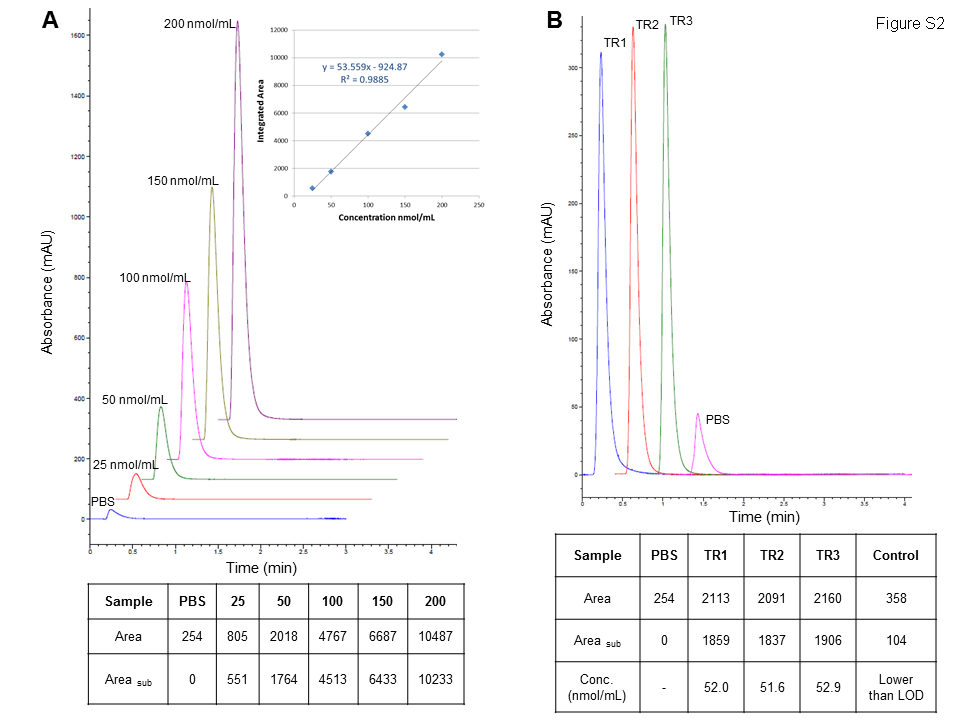

Supplement: Figure S2 — HPLC-based quantification of VS1 in mouse blood. (A) Peak absorbance at 210 nm was measured for VS1-biotin standards ranging in concentration of biotin from 25–200 nmol/ml (corresponds to 0.1375–1.1 mg/ml of biotinylated VS1-NH2). The table below the graph provides the integrated area under the peak for each standard, as well as those values with the blank subtracted (Areasub). The inset graph shows the standard curve generated from the linear relationship of integrated area under the peak and concentration of biotin. (B) Peak absorbance at 210 nm was measured for three technical replicates (TR) of VS1-biotin isolated from mouse blood. The table below the graph reports integrated area under the peak for each TR, those values with the blank subtracted (Areasub), and the estimated concentration based on the standard curve. (TIF) [file ppat.1003757.s002.tif]

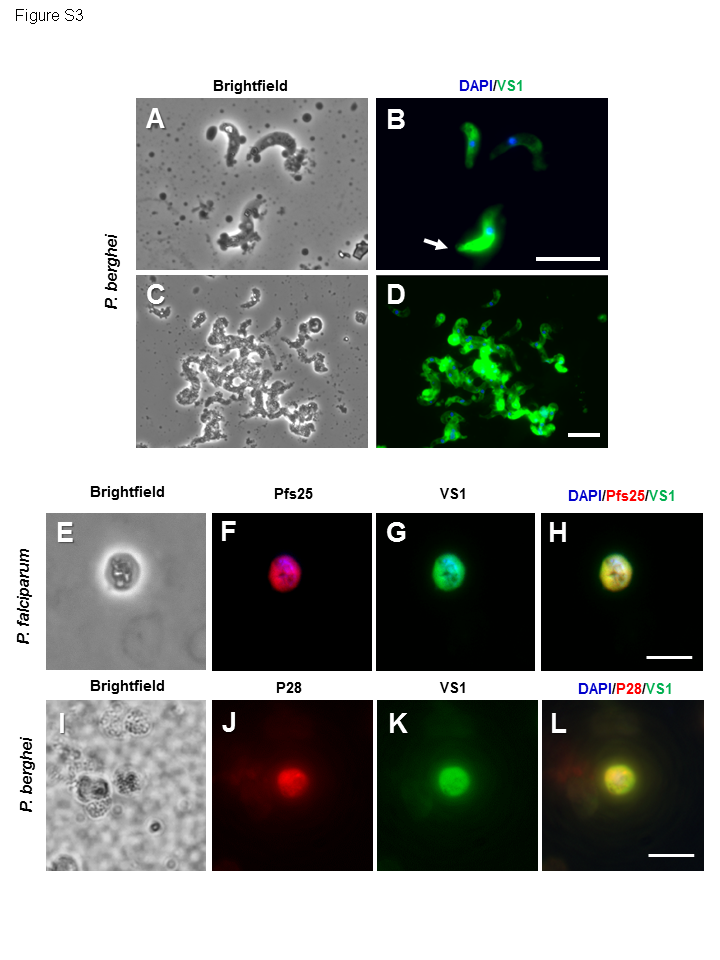

Supplement: Figure S3 — Additional microscopy images demonstrating VS1 binding to mosquito-stage parasites. (A–D) Brightfield and immunofluorescence microscopy images of VS1 staining patterns of permeabilized wild type ookinetes from P. berghei. Each pair of images depicts brightfield (A, C), followed by staining with VS1 (green) merged with DAPI nuclear staining (blue) (B, D). The arrow in panel B highlights greater VS1 staining intensity apically. Size bar = 10 µm. (E–L) Microscopy images of VS1 staining patterns of permeabilized round cells from wild type P. falciparum (E–H) and P. berghei (I–L). Each row of images depicts brightfield (E,I), followed by staining with Pfs25 (red) (F) or P28 (red) (J), VS1 (green) (G, K), and the merged image of VS1, PfS25/P28, and DAPI nuclear staining (blue). Size bar = 10 µm. (TIF) [file ppat.1003757.s003.tif]

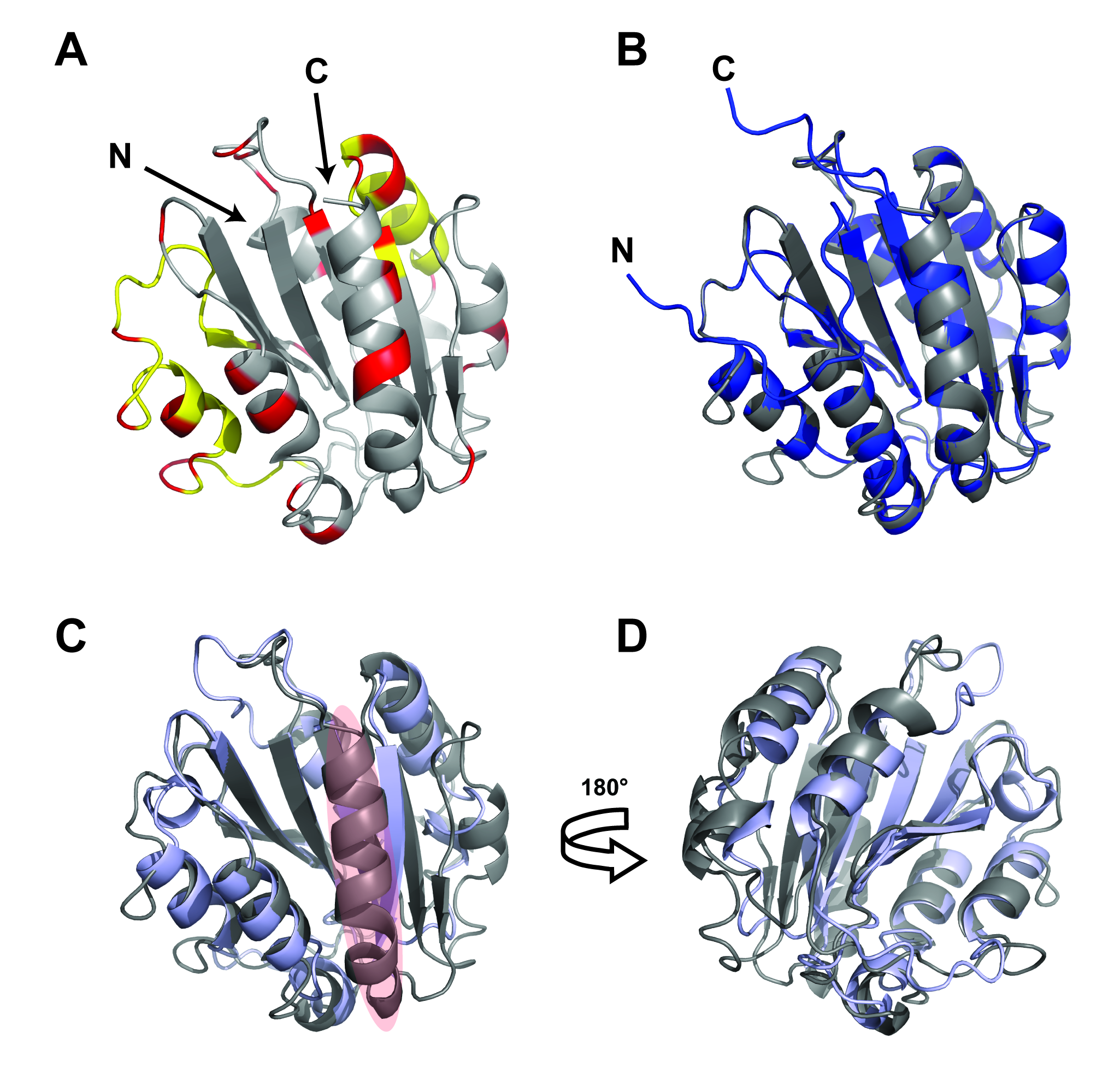

Supplement: Figure S4 — Plasmodium gametocytes are not stained with biotinylated VS1. (A–D) Brightfield and fluorescence microscopy images of VS1 staining patterns for non-permeabilized wild type gametocytes from P. berghei (A–B) and P. falciparum (C–D). Each pair of images depicts brightfield (A, C), followed by staining with VS1 (red) merged with DAPI nuclear staining (blue) (B, D). Black and white arrows denote the location of the gametocytes. Size bar = 10 µm. (TIF) [file ppat.1003757.s004.tif]

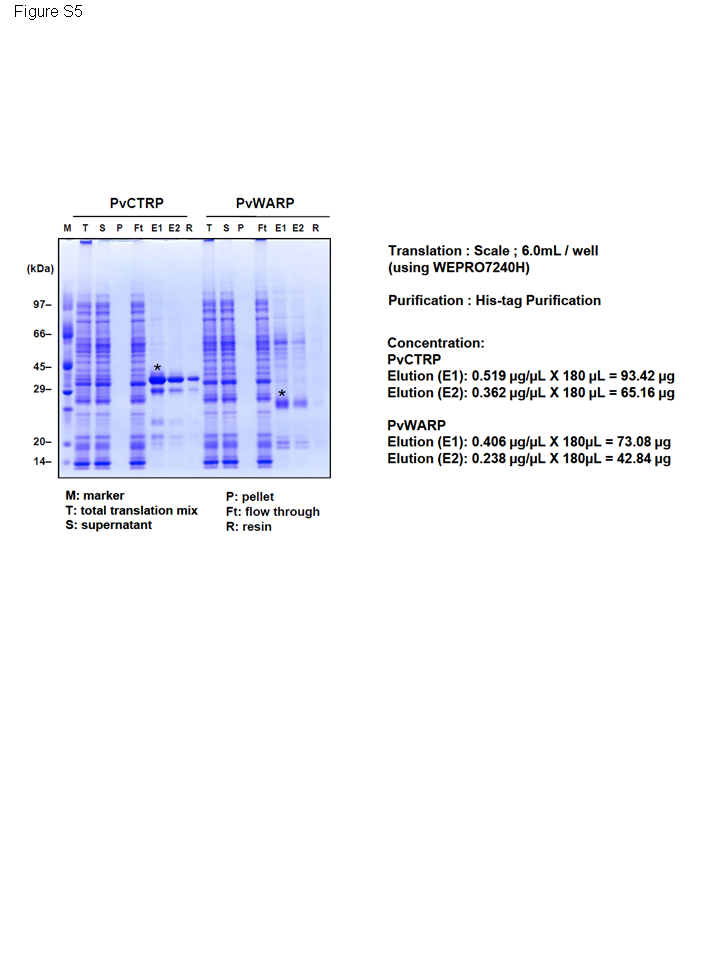

Supplement: Figure S5 — SDS-PAGE gel demonstrating the wheat germ cell free system for soluble expression of His-tagged recombinant Plasmodium vivax CTRP and WARP. Asterisks denote the sample material used in subsequent assays. M: molecular weight marker. T: Total translation mix. S: Supernatant. P: Pellet. Ft: Flow through. Ne: Non-reducing condition elution. Re: Reducing condition elution. R: Resin. Scale: 6 µl/well. Concentration: PvCTRP (162 µg in 200 µl) and PvWARP (58 µg in 200 µl). (TIF) [file ppat.1003757.s005.tif]

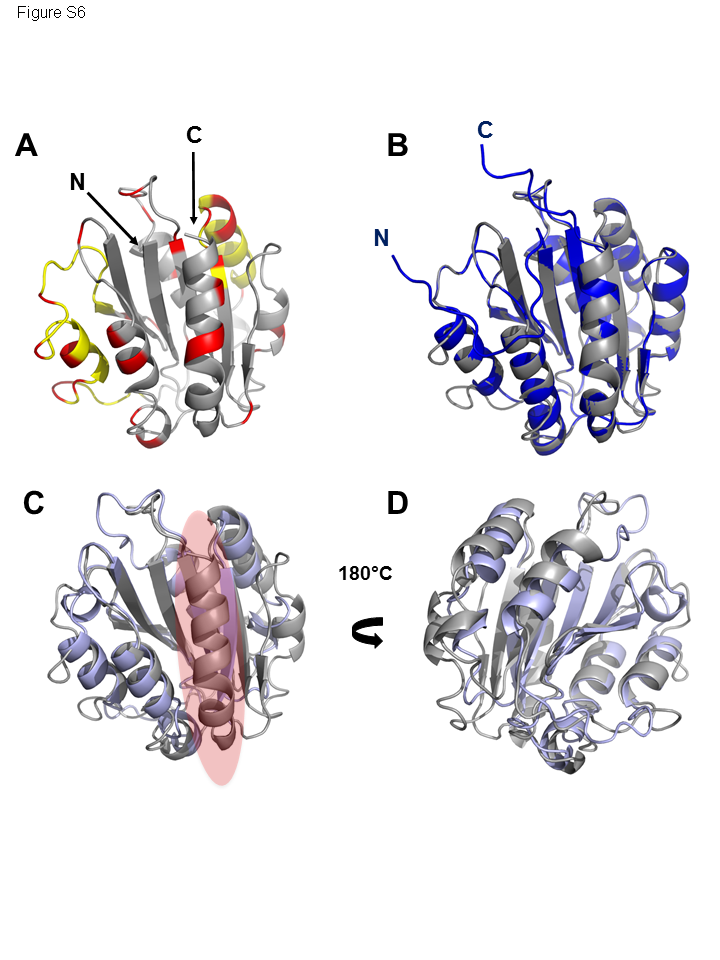

Supplement: Figure S6 — VS1, a proof of concept molecular mimic of apical midgut chondroitin sulfate glycosaminoglycan binds to Plasmodium Circumsporozoite and Thrombospondin Related Anonymous Protein Related Protein (CTRP). (A) Homology model of Plasmodium falciparum CTRP (Model 1) based on the crystal structure of human von Willebrand Factor A1 (vWFA1) domain (PDB: 1AUQ). (B) Superposition of the PfCTRP Homology (Model 1, gray) on the vWFA1 crystal structure (1AUQ, blue). Structures/models for (B) and (C) are in same orientation as Figure 5 (B–E). Note that the structure and model are similar, with the exception of a few loops and the N- and C-termini. (C) Superposition of Model 1 (gray) and Model 2 (Fig. 5 (D–E), based on Toxoplasma MIC2 structure, 2XGG) and the same image rotated 180° in (D). The main difference between Model 1 and Model 2 is shaded in red, which is an extra helix modeled in Model 1 but is missing in Model 2. (TIF) [file ppat.1003757.s006.tif]
