# Supplementary material for: A Small Molecule Glycosaminoglycan Mimetic Blocks Plasmodium Invasion of the Mosquito Midgut
Source: PLoS Pathog. 2013 Nov 21;9(11):e1003757. doi: 10.1371/journal.ppat.1003757 (PMC3836724; doi:10.1371/journal.ppat.1003757)
Supplement: Table S2 — Male gametocyte exflagellation data from Plasmodium berghei ANKA 2.34-infected mice pre- and post-injection with either PBS (carrier-only control), PVP (non-sulfated polymer control), or VS1-3000. Exflagellation data from groups of 5 mice are presented in three columns per treatment group. The first two columns show the average number of exflagellation centers per 40× field from five fields recorded both pre- and post-injection for individual mice. The third column provides the P-value for a comparison at the group level of the average number of exflagellation centers in pre- versus post-injection mice. Note that a different set of mice was used for each treatment (i.e., Mouse 1 is not the same between PBS, PVP, and VS1 treatment groups). (DOCX) [file ppat.1003757.s008.docx]

| **Mouse** | **PBS^a^**  **(Pre)** | **PBS**  **(Post)** | ***P*-value^b^** | **PVP**  **(Pre)** | **PVP**  **(Post)** | ***P*-value** | **VS1**  **(Pre)** | **VS1**  **(Post)** | ***P*-value** |
| --- | --- | --- | --- | --- | --- | --- | --- | --- | --- |
| **1** | 1.4 | 1.2 |  | 2.4 | 2.2 |  | 1.6 | 1.8 |  |
| **2** | 1.4 | 1.6 |  | 1.2 | 2.0 |  | 0.8 | 3.6 |  |
| **3** | 2.2 | 2.0 |  | 2.2 | 3.4 |  | 3.2 | 2.4 |  |
| **4** | 3.6 | 4.0 |  | 2.4 | 3.0 |  | 1.6 | 4.6 |  |
| **5** | 5.4 | 3.2 |  | 4.0 | 4.2 |  | 1.6 | 5.0 |  |
| **Mean**  **(SE)** | 2.8 (0.8) | 2.4 (0.5) | 0.3980 | 2.4 (0.4) | 3.0 (0.4) | 0.1169 | 1.8 (0.4) | 3.1 (0.6) | 0.0006 |

^a^For each treatment group, the number of exflagellation centers per 40X field was determined pre- and post-injection (iv) of the treatment.

^b^Level of significance from two-way analysis of variance (ANOVA); note that VS1 had a significant effect but in the opposite direction one would predict based on the observed oocyst intensity in the mosquitoes dissected from this experiment (VS1-3,000, replicate 1 in Table 1).

**Table S2. Male gametocytes exflagellation data from *Plasmodium berghei* ANKA 2.34 *-*infected mice pre- and post-injection with either PBS (carrier-only control), PVP (non-sulfated polymer control), or VS1-3000.**
