# Supplementary material for: A Small Molecule Glycosaminoglycan Mimetic Blocks Plasmodium Invasion of the Mosquito Midgut
Source: PLoS Pathog. 2013 Nov 21;9(11):e1003757. doi: 10.1371/journal.ppat.1003757 (PMC3836724; doi:10.1371/journal.ppat.1003757)
Supplement: Text S1 — This file contains the materials and methods corresponding to the supplementary Figure S1 (Capillary Electrophoresis Laser Induced Fluorescence analysis of midgut BBMV Chondroitin sulfate GAGs), Figure S2 (Determination of VS1 concentration in mouse blood), and Figure S4 ( Plasmodium gametocytes immunostaining). (DOCX) [file ppat.1003757.s009.docx]

**Supplementary Material for the Article**

**A small molecule glycosaminoglycan mimetic blocks *Plasmodium* invasion of the mosquito midgut**

Derrick K. Mathias^1,6^, Rebecca Pastrana-Mena^1,6^, Elisabetta Ranucci^2^, Dingyin Tao^1^, Paolo Ferruti^2^, Corrie Ortega^1^, Gregory O. Staples^3^, Joseph Zaia^3^, Eizo Takashima^4^, Takafumi Tsuboi^4^, Natalie A. Borg^5^, Luisella Verotta^2^, Rhoel R. Dinglasan^1,^*

^1^W. Harry Feinstone Department of Molecular Microbiology and Immunology, Malaria Research Institute, Johns Hopkins Bloomberg School of Public Health, 615 North Wolfe Street, Baltimore, MD 21205, USA; ^2^Department of Chemistry, University of Milan, via Camillo Golgi 19, 20133 Milan, Italy; ^3^Department of Biochemistry and Center for Biomedical Mass Spectrometry, Boston University School of Medicine, Boston, MA, 02118, USA; ^4^ Division of Malaria Research, Proteo-Science Center, Ehime University, 3 Bunkyo-cho, Matsuyama, Ehime 790-8577, Japan; and ^5^Department of Biochemistry and Molecular Biology, School of Biomedical Sciences, Monash University, Victoria 3800, Australia.

^6^Authors contributed equally.

1. **Materials and Methods**

**Capillary Electrophoresis Laser Induced Fluorescence analysis of midgut BBMV Chondroitin sulfate GAGs.** Brush border microvilli vesicle preparations from two replicate samples of 1,500 female *An. gambiae* KEELE midguts were prepared as previously described [1]. Samples were prepared and digested with chondroitinase ABC; and the resultant disaccharides from the digest were derivatized with 2-aminoacridone, followed by analysis using capillary electrophoresis with laser induced fluorescence detection as described [2].

**Immunofluorescence microscopy assays.** Gametocyte samples were fixed with 4% paraformaldehyde and prepared for fluorescence microscopy by washing three times with PBS. After the washes, samples were blocked with 3% BSA for 30 minutes. The samples were then incubated with biotinylated-VS1 for 1 hr at RT. Cells were washed as before and incubated with Dylight 594 conjugated Streptavidin (Thermo) for 1 hr at RT. Following incubation, the cells were washed three times with PBS, resuspended in PBS, spotted on slides and allowed to air dry. Samples were mounted using Slow Fade Gold antifade reagent with DAPI (Molecular Probes). Samples were examined with SPOT software using a Nikon Upright E800 microscope.

**Determination of VS1 concentration in mouse blood.** Prior to analyzing mouse blood, a standard curve was generated using dilutions of biotinylated VS1 in PBS ranging from 25 to 200 nmol/ml. The standard with the highest concentration was diluted from a stock solution quantified using the Quant Tag Biotin Kit (Vector Labs) following the manufacturer’s protocol for the microplate assay and then used to make the other standards via serial dilution. Note that a concentration of 200 nmol/ml of biotin corresponds to 1.1 mg/ml of biotinylated VS1 when using the average molecular weight of VS1-NH_2_ (Figure 1A) plus the biotin molecule used in the biotinylation reaction (EZ-Link Sulfo-NHS-LC-LC-Biotin, Thermo Scientific). To generate the curve, 12 µl of each standard was directly injected into the HPLC UV detector (Agilent 1260 series) at a flow rate of 0.3 ml/min with 10 mM Tris-HCl, pH 7.5, as the mobile phase. The peak absorbance at 210 nm was measured for each standard and quantified as the integrated area (0.1 min to 0.6 min) under the peak.

To confirm that VS1 was present in the blood of experimental mice, and therefore available for ingestion by mosquitoes, a *P. berghei* (ANKA 2.34) infected female mouse (Swiss Webster, 22.3 g) was intravenously injected with 250 µg of VS1-biotin in 200 µl of PBS. The compound was allowed to circulate in the bloodstream for 15 minutes followed by euthanization and collection of blood via cardiac puncture. To isolate the VS1 compound (which we assume would be in the plasma), 500 µl of whole blood was centrifuged at 2000 x g for 10 minutes to pellet the RBCs and the plasma fraction was carefully drawn off and transferred to a 1.5 ml tube by pipet. For analysis, a 100 µl sample of plasma was removed, mixed with 4 volumes of cold acetone (-20°C), and incubated overnight at -20°C. The sample was then centrifuged for 10 minutes at 15,000 x g at 4°C to pellet the acetone-precipitated proteins. The supernatant was transferred to a new tube and centrifuged under a vacuum to near dryness to remove the acetone and concentrate the sample. Following centrifugation, the volume was brought up to 100 µl with PBS and added to an equal volume of Protein A/G MagBeads (GenScript) bound and cross-linked with 250 µg of anti-biotin mAb (Invitrogen). The sample was incubated with the beads at room temperature for one hour with gentle agitation. The beads were then collected with a magnetic separator and washed three times with PBS. Biotinylated VS1 was then eluted from the beads by incubating for 5 minutes with 100 µl of IgG Elution Buffer (Thermo Scientific) three times, immediately neutralizing each eluate with 10 µl 1 M Tris-HCl, pH 8.5. The eluates were pooled and buffer exchanged into PBS using diafiltration with a 0.5 ml centrifugal filter (Amicon, 3,000 Da MWCO). The buffer exchanged and concentrated sample (total volume of 56 µl) was then analyzed using the HPLC UV detector as described above for generating the standard curve.

The mean concentration of VS1-biotin purified from the plasma sample was estimated to be 50.6 nmol/ml based on the mean area under the peak of three technical replicates and the linear relationship between integrated area and concentration (i.e., the inset standard curve in Figure S4A). Multiplying this concentration by the total volume of purified VS1-biotin following buffer exchange yields the total amount of biotinylated VS1 purified from the plasma sample: 50.6 nmol/ml * 0.056 ml = 2.83 nmol. From this estimate, the total amount circulating in the bloodstream can be estimated by taking the product of the amount purified and the dilution factor of the plasma sample: 2.83 nmol * (1300 µl total plasma volume^[[1]](#footnote-1)^ divided by 100 µl plasma sample volume) = 36.79 nmol. Using the same standard curve and HPLC methodology, the concentration of VS1-biotin initially injected into the mouse was verified to be 228 nmol/ml (approximately 1.25 mg/ml). Given the 200 µl injection volume, the total amount expected in the bloodstream was 45.6 nmol (or 250 µg). Our empirical estimate of 36.79 nmol indicates that we recovered 80.7% of the biotinylated compound from our plasma sample.

1. **Table**

1. **References**
2. Dinglasan RR, Kalume DE, Kanzok SM, Ghosh AK, Muratova O, et al. (2007) Disruption of *Plasmodium falciparum* development by antibodies against a conserved mosquito midgut antigen. Proc Natl Acad Sci USA 104(33): 13461-13466.
3. Hitchcock AM, Bowman MJ, Staples GO, Zaia J. (2008) Improved workup for glycosaminoglycan disaccharide analysis using CE with LIF detection. Electrophoresis 29(22): 4538-4548.

1. Based on the weight of the mouse (22.3 g) and the blood volume-to-weight ratio of 2000 µl per 24 g, the blood volume of this mouse was estimated to be 1858 µl. Anemia caused by a *P. berghei* infection reduced the hematocrit in this mouse to approximately 30%. Therefore, the total plasma volume was estimated to be 70% of the blood volume, or 1300 µl. [↑](#footnote-ref-1)
